# Ultrasensitive In Vivo Imaging of Adoptive Immune Cell Distribution and Expansion Using Second Near-Infrared Conjugated Oligoelectrolyte Probes

**DOI:** 10.34133/research.1342

**Published:** 2026-06-26

**Authors:** Shengnan Yuan, Qingshuang Li, Xi Kang, Yingying Meng, Pengke Liu, Pengfei Zhang, Jin Zhang, Dehong Hu, Duyang Gao, Caoyun Ju, Xiuqi Li, Can Zhang, Hairong Zheng, Nuernisha Alifu, Cheng Zhou, Zonghai Sheng

**Affiliations:** ^1^ Research Center for Advanced Detection Materials and Medical Imaging Devices, Institute of Biomedical and Health Engineering, Shenzhen Institutes of Advanced Technology, Chinese Academy of Sciences, Shenzhen 518055, P. R. China.; ^2^ State Key Laboratory of Biomedical Imaging Science and System, Shenzhen 518055, P. R. China.; ^3^ Translation Innovation Center, Shenzhen Bay Lab, Shenzhen 518132, P. R. China.; ^4^ Institute of Polymer Optoelectronic Materials and Devices, Guangdong Basic Research Center of Excellence for Energy & Information Polymer Materials, State Key Laboratory of Luminescent Materials and Devices, School of Materials Science and Engineering, South China University of Technology, Guangzhou 510640, P. R. China.; ^5^ State Key Laboratory of Natural Medicines and Jiangsu Key Laboratory of Drug Discovery for Metabolic Diseases, Center of Advanced Pharmaceuticals and Biomaterials, China Pharmaceutical University, Nanjing 210009, P. R. China.; ^6^ State Key Laboratory of Pathogenesis, Prevention and Treatment of High Incidence Diseases in Central Asia, School of Medical Engineering and Technology & Technology Innovation and Translational Service Center, Xinjiang Medical University, Urumqi 830054, P. R. China.

## Abstract

Monitoring adoptive cell therapy in solid tumors is critical for evaluating treatment efficacy and guiding clinical medication but is also hindered by poor sensitivity, high background signals, and disruptions of therapeutic functions in existing techniques. In this study, a membrane-mimicking conjugated oligoelectrolyte with second near-infrared (NIR-II) fluorescence, conjugated oligoelectrolytes-benzobisthiadiazole (COE-BBT), is applied for the first time to label and track chimeric antigen receptor (CAR)-engineered natural killer (CAR-NK) cells and T (CAR-T) cells in vivo. COE-BBT stably embeds in lipid bilayers through combined electrostatic and hydrophobic interactions, resists membrane crossing, and supports long-lasting labeling with a lighting-up property. The optimized labeling approach achieves high sensitivity, enabling the detection of as few as ~20 labeled cells in vitro and ~50 cells in vivo under NIR-II imaging. In orthotopic and subcutaneous glioma models, NIR-II fluorescence imaging enables continuous tracking of CAR-NK and CAR-T cell proliferation, migration, tumor homing, and blood–brain barrier penetration for up to 14 d posttransfer as the fluorescence signal is enhanced during proliferation, without compromising cell viability or cytotoxic function. The COE-BBT probe also exhibits favorable biosafety, underscoring its translational potential as a robust imaging strategy to improve solid tumor adoptive cell therapy monitoring and clinical guidance of therapeutic dosing.

## Introduction

Adoptive cell therapy (ACT), including chimeric antigen receptor (CAR) T (CAR-T) cell therapy, CAR natural killer (CAR-NK) cell therapy, and T-cell receptor-engineered T cell therapy, has emerged as a pioneering approach in oncology [1–3]. Through ex vivo genetic modification and expansion of patients’ immune cells, ACT enables precise recognition of tumor-specific antigens and potent cytotoxicity [4]. Although ACT has demonstrated breakthrough efficacy in hematologic cancers, its clinical translation in solid tumors remains constrained by multiple bottlenecks [1,5]. Among them, the lack of real-time visualization of ACT cell dynamics in vivo is a key obstacle [6]. Precise delineation of the in vivo fate of ACT cells, including biodistribution, proliferation, migration, and tumor homing, constitutes a core criterion for advancing living-cell therapies from bench to bedside [7]. Such a resolution provides a direct basis for optimizing treatment regimens, adjusting doses, and managing toxicity [8,9]. Furthermore, it informs strategies to overcome barriers characteristic of solid tumors, notably inadequate immune cell infiltration and an immunosuppressive tumor microenvironment [9]. Therefore, strengthening fate mapping of ACT cells is essential to accelerate clinical translation and improve therapeutic efficacy in solid tumors.

To date, several approaches have been used for real-time in vivo tracking of ACT cells, including radiolabeling [7,10,11], photoacoustic imaging [12], and magnetic resonance imaging [13,14]. However, these approaches are constrained by ionizing radiation, complicated engineering progress, high background signals, and limited sensitivity for cell detection [15]. Second near-infrared (NIR-II; 1,000 to 1,700 nm) fluorescence imaging has emerged as an in vivo modality with a high contrast, reduced tissue scattering, minimal background signals, and improved tissue penetration [15–17]. It has been widely applied to the imaging and operation of deep-seated solid tumors [18,19]. These features suggest that NIR-II imaging could serve as an effective platform for quantifying CAR cell accumulation in solid tumors. Consequently, various fluorescence probes have been developed for in vivo CAR-T cell tracking, including Food and Drug Administration-approved indocyanine green (ICG) [20], classical small-molecule membrane dyes derived from cyanine or BODIPY scaffolds [21], and nanoparticle-based probes for preclinical research [22,23]. Nevertheless, existing probes are limited by short signal persistence and membrane dissociation due to unstable cell labeling [24]. In addition, the corresponding labeling requires probe internalization by the labeled cells [25]. Therefore, improved NIR-II probes are urgently required to enable longitudinal in vivo monitoring to support the clinical application of CAR cells.

**Fig. 1.**
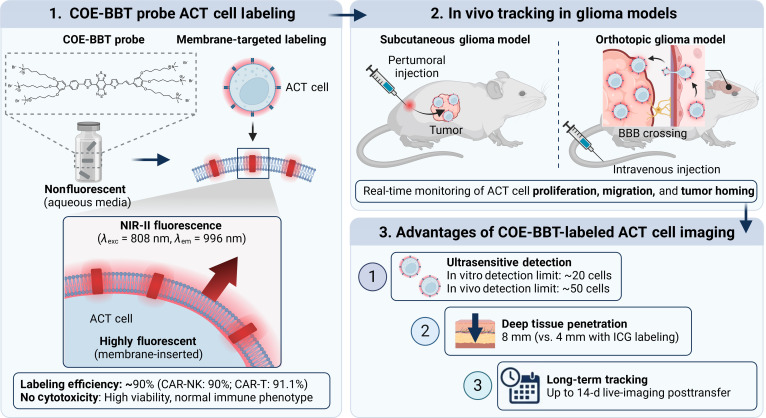
Schematic illustration of conjugated oligoelectrolytes-benzobisthiadiazole (COE-BBT)-mediated second near-infrared (NIR-II) fluorescence labeling and in vivo tracking of adoptive cell therapy (ACT) cells in glioma models. The platform has significant advantages, including ultrasensitive detection, improved deep-tissue imaging penetration, and long-term tracking after cell transfer. The image was created using BioRender.com and is used with permission.

Conjugated oligoelectrolytes (COEs) are a category of amphiphilic materials that feature a linear hydrophobic π-conjugated backbone with ionic functional side chains at both terminals [26]. The membrane-mimicking molecular platforms of COEs were developed to exhibit increased fluorescence intensity in a lipophilic environment, which leverages tunable optical properties while retaining stable membrane association [27,28]. Based on these properties, we recently extended the optical window of a COE probe into the NIR-II range by inserting a benzobisthiadiazole (BBT)-based donor–acceptor–donor fragment. In our previous studies, conjugated oligoelectrolytes-benzobisthiadiazole (COE-BBT) was explored for tumor-cell-associated applications, including observation of tumor cell proliferation and labeling of cancer-cell-derived extracellular vesicles [29,30]. To further expand its applicability to monitoring cell-based therapeutic approaches, the COE-BBT probe was used for the first time in this study to track CAR-engineered immune cells in vivo. Using an NIR-II live-imaging system, we dynamically tracked the proliferation, migration, and tumor-homing dynamics of COE-BBT-labeled CAR-NK and CAR-T cells in glioma-bearing mice. This work establishes a reliable strategy for real-time, quantitative in vivo monitoring of CAR cells, enabling highly sensitive detection and guiding future dosing regimens in CAR-immune cell therapies (Fig. 1).

## Results and Discussion

### In vivo CAR-immune cell labeling with the COE-BBT probe

**Fig. 2.**
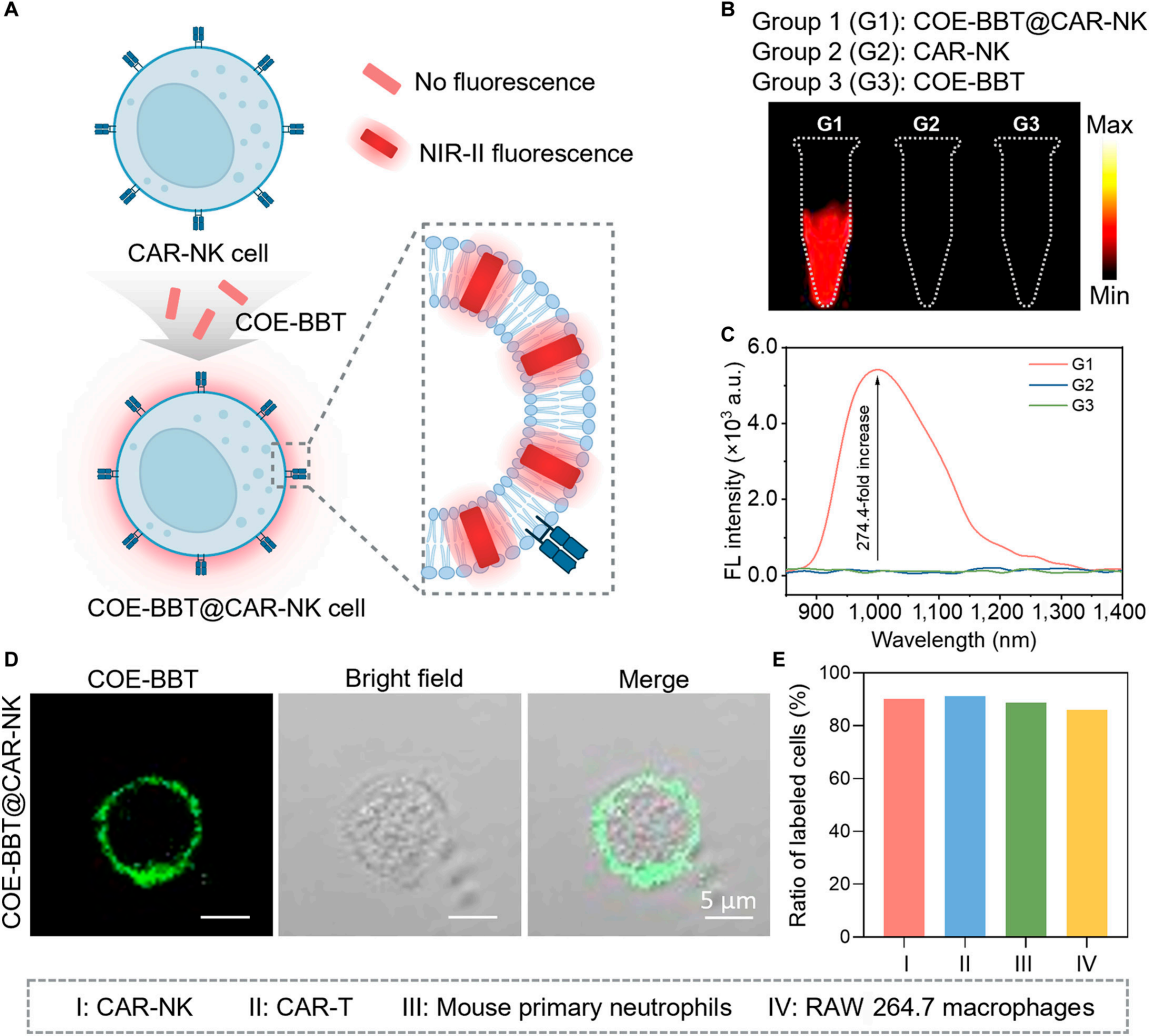
Cell membrane labeling with the COE-BBT fluorescence probe. (A) Schematic of conjugated oligoelectrolytes-benzobisthiadiazole (COE-BBT) cell membrane labeling. The image was created using BioRender.com and is used with permission. (B) Second near-infrared (NIR-II) fluorescence image of COE-BBT-labeled chimeric antigen receptor (CAR)-engineered natural killer (CAR-NK) cells (G1), CAR-NK cells free of labeling (G2), and free COE-BBT in phosphate-buffered saline (PBS) (G3) under optimized labeling conditions (*λ*_exc_ = 808 nm, 1,200-nm long-pass filter, exposure time, 1,500 ms). (C) Fluorescence emission spectrum of COE-BBT-labeled CAR-NK cells (G1), CAR-NK cells free of labeling (G2), and free COE-BBT in PBS (G3). (D) Confocal image of COE-BBT-labeled CAR-NK cells. Scale bar, 5 μm. (E) Flow cytometry analysis of COE-BBT labeling efficiency in CAR-NK (I), CAR-engineered T (CAR-T) (II), mouse primary neutrophils (III), and RAW 264.7 macrophages (IV).

The labeling capability of the COE-BBT probe was first evaluated on CAR-NK cells, which were established from the human NK-92 MI cell line and characterized in our lab (Fig. 2A and Fig. S1). COE-BBT alone was essentially nonemissive in phosphate-buffered saline (PBS) because its charge-transfer excited state is strongly stabilized in the highly polar aqueous environment, thereby promoting nonradiative decay. Upon insertion into the lipid bilayer, the BBT-containing conjugated core is transferred into a conformationally restricted and less polar environment, thereby reducing solvent-induced quenching and enhancing fluorescence [30,31]. Indeed, the NIR-II fluorescence intensity of COE-BBT in PBS solution was undetectable. By contrast, COE-BBT-labeled CAR-NK (COE-BBT@CAR-NK) cells exhibited bright NIR-II emission under optimized conditions (Fig. S2 and Fig. 2B). Fluorescence spectroscopy detected a maximal emission wavelength of 996 nm for COE-BBT after cell labeling, consistent with previous research [29]. Quantitatively, the fluorescence intensity for COE-BBT@CAR-NK cells increased by 274.4-fold relative to that for COE-BBT alone in PBS, indicating efficient and stable labeling of CAR-NK cells (Fig. 2C). Confocal microscopy revealed membrane localization of the COE-BBT probe, with intense peripheral fluorescence on CAR-NK cells (Fig. 2D). To demonstrate the universality of the COE-BBT probe, the optimized COE-BBT labeling protocol was extended to human-derived CAR-T cells, mouse primary neutrophils, and RAW 264.7 macrophages. Flow cytometry analysis revealed a remarkable labeling efficiency of COE-BBT for CAR-NK (90.1%), CAR-T (91.1%), neutrophils (88.8%), and macrophages (86.0%) (Fig. 2E). These results establish COE-BBT as a high-efficiency NIR-II probe that robustly labels diverse immune cells on their membranes.

### Molecular dynamics simulation of the COE-BBT–membrane interaction

**Fig. 3.**
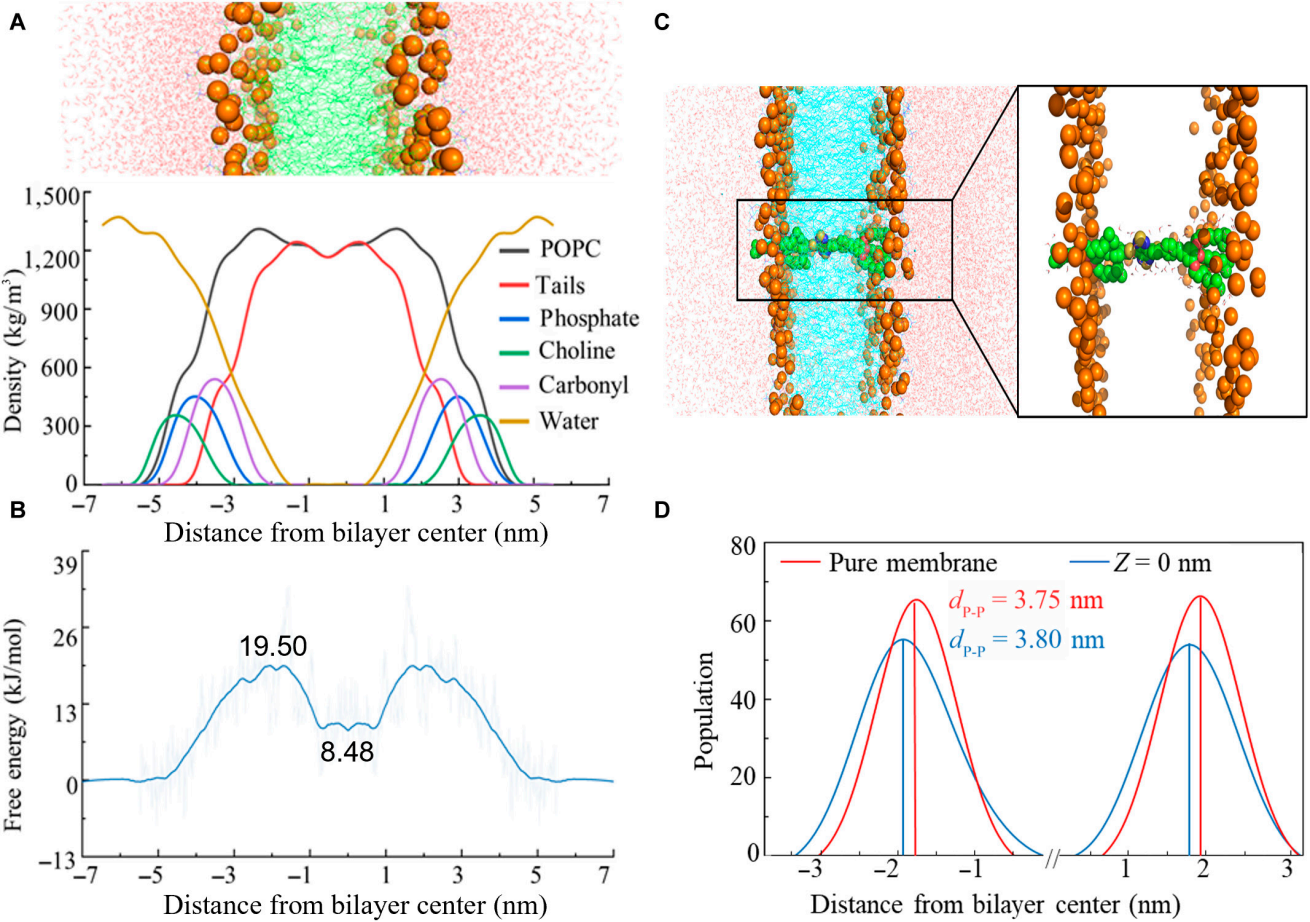
Molecular dynamics (MD) simulation study on the interaction of conjugated oligoelectrolytes-benzobisthiadiazole (COE-BBT) and the cellular membrane. (A) Snapshot and the partial density profiles of the equilibrated lipid membrane. (B) Free-energy profiles of COE-BBT. The free energy was set to zero in bulk water. (C) Global snapshots of COE-BBT in the phospholipid bilayer (at the position 0 Å from the center of the phospholipid bilayer). (D) Distribution of the phosphate groups of the pure membrane and the membrane with COE-BBT inserted at the selected *Z*-position. The membrane thickness is labeled as *d*_P-P_.

To gain further insight into the membrane-labeling behavior of COE-BBT, molecular dynamics (MD) simulations were performed. To assist in describing free-energy profiles, we constructed a simplified 1-palmitoyl-2-oleoyl-*sn*-glycero-3-phosphocholine (POPC-based phospholipid bilayer using partial density profiles of the lipid membrane (Fig. 3A). The membrane center is taken as the reference, with the *Z*-position defined as *Z* = 0 Å. The free-energy profile of COE-BBT through the lipid membrane is illustrated in Fig. 3B. A sharp increase in the free energy of the small molecule was observed as it approached the phospholipid bilayer, which may be attributed to the electrostatic repulsion exerted by the positively charged choline groups on the membrane surface on the positively charged moieties of the small molecule. Subsequently, the free energy decreased rapidly once the positively charged moieties of the small molecule were fully inserted into the membrane, reaching a minimum in the phosphate and carbonyl regions. This phenomenon could be explained by the strong electrostatic attraction between the small positively charged molecule and the phosphate and carbonyl moieties, both of which carry a strong negative charge; however, the migration energy remained relatively high at this stage. Specifically, the free energy of the small molecule peaked at 33.109 kJ/mol at the position of *Z* = −2.0 nm. As the small molecule moved toward the hydrophobic tails at the membrane center, its free energy decreased slightly, reaching a local minimum at *Z* = −1.0 nm. The average free-energy value of the small molecule throughout the entire transmembrane process was approximately 10.799 kJ/mol. Overall, the small molecule exhibited a relatively high free-energy barrier during transmembrane translocation, indicating that it is difficult for this molecule to leak across the membrane spontaneously. At the global minima of the free-energy profiles, COE-BBT inserted vertically into the phospholipid membrane with its positively charged headgroups located in the headgroup region of the lipid membrane and are surrounded by the negatively charged phosphate groups, while the hydrophobic BBT moieties of the molecular lie inside the hydrophobic core of the lipid membrane, which caused the membrane thickness *d*_P–P_ increase by 0.05 nm (Fig. 3C and D). The intermolecular interactions between COE-BBT and surrounding POPC molecules showed that distinct van der Waals interactions are observed between the small molecule and the surrounding hydrophobic tail chains of POPC (Fig. S3). From the above analysis, we infer that COE-BBT has a favorable tendency to adopt a membrane-inserted configuration, driven by electrostatic interactions between its cationic headgroups and phospholipid phosphate groups, together with hydrophobic interactions of the BBT core. These simulations provide mechanistic support for membrane association. However, as a simplified model, the POPC bilayer is weak in capturing the compositional complexity of mammalian plasma membranes. Future studies using asymmetric multicomponent membranes containing cholesterol could further validate the insertion behavior of COE-BBT.

### Function characterization of COE-BBT-labeled CAR-NK cells

Having established a robust labeling protocol, we assessed whether COE-BBT labeling perturbs CAR-NK cell functions. Compared with controls, COE-BBT-labeled NK cells showed identical viability and proliferation rates (Fig. S4a and b). Bright-field imaging revealed no observable morphological alterations in labeled cells, which retained the characteristic aggregated growth (Fig. S4c). To determine the impact of COE-BBT labeling on the CAR-NK cell phenotype, flow cytometry was performed to analyze canonical NK markers. The fractions of CD3^−^CD56^+^ and CD3^−^CD16^+^ cells were preserved after labeling, indicating that lineage identity was maintained (Fig. S4d and e). Annexin V–fluorescein isothiocyanate (FITC)/propidium iodide (PI) staining revealed no increase in the apoptosis of labeled cells (Fig. S4f). Enzyme-linked immunosorbent assay (ELISA) further confirmed consistent secretion of the cytokines tumor necrosis factor-α (TNF-α), interferon-γ (IFN-γ), and interleukin-2 (IL-2) following COE-BBT labeling (Fig. S4g). Collectively, these data demonstrate that COE-BBT labeling is functionally inert for CAR-NK cells.

### Imaging depth and sensitivity of COE-BBT@CAR-NK cells

**Fig. 4.**
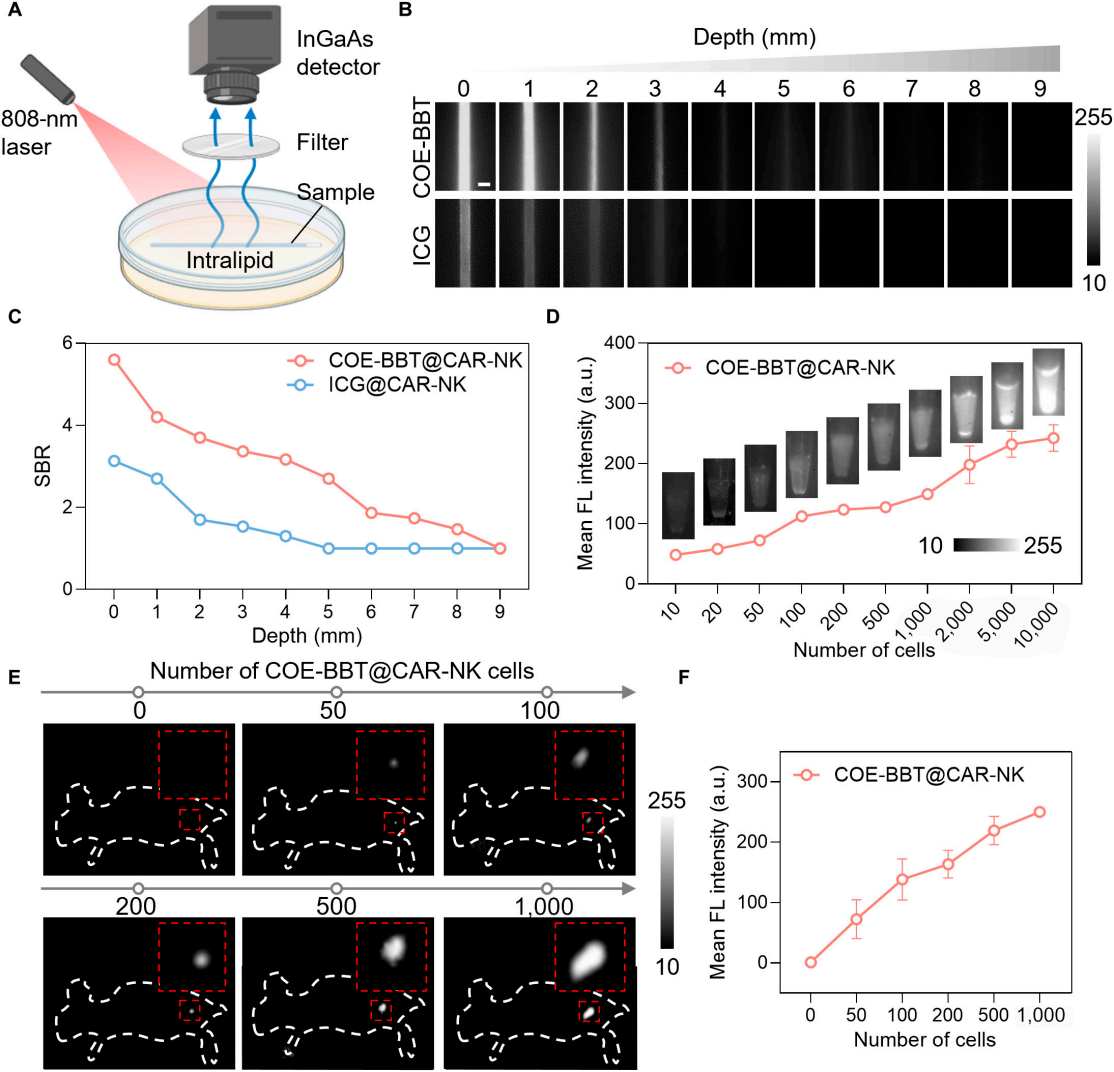
Characterization of imaging depth and sensitivity of conjugated oligoelectrolytes-benzobisthiadiazole (COE-BBT)-labeled chimeric antigen receptor (CAR)-engineered natural killer (CAR-NK) cells. (A) Schematic of the second near-infrared (NIR-II) imaging system. The image was created using BioRender.com and is used with permission. (B) NIR-II fluorescence (FL) images (*λ*_exc_ = 808 nm) of a glass capillary filled with CAR-NK cells labeled with COE-BBT or indocyanine green (ICG) in 1% intralipid varied in depth (0 to 9 mm). Scale bar, 1 mm. Images captured utilizing a 1,200-nm long-pass filter, 1,200-ms exposure time. (C) Mean FL intensity quantification of COE-BBT@CAR-NK and ICG@CAR-NK cells in panel (B). Data represent mean ± SD, *n* = 3. (D) NIR-II FL images (*λ*_exc_ = 808 nm) and the mean FL intensity quantification of a test tube filled with COE-BBT@CAR-NK cells with varied cell numbers. Images captured utilizing a 1,200-nm long-pass filter, 1,500-ms exposure time. Data represent mean ± SD, *n* = 3. (E) NIR-II FL images (*λ*_exc_ = 808 nm) and (F) mean FL intensity quantification of nude mice subcutaneously injected with COE-BBT@CAR-NK with varied cell numbers. Images captured utilizing a 1,000-nm long-pass filter, 500-ms exposure time. Data represent mean ± SD, *n* = 3.

Next, we assessed the maximum penetration depth and imaging sensitivity of COE-BBT-labeled CAR-NK cells under NIR-II fluorescence imaging, compared with ICG, a clinically approved fluorophore utilized for cell tracking [16]. The penetration depth of COE-BBT-labeled cells was quantified under in vitro conditions with 1% intralipid (Fig. 4A). COE-BBT@CAR-NK cells produced NIR-II emission that traversed up to 8 mm. In comparison, ICG-labeled CAR-NK cells penetrated only ~4 mm of the medium (Fig. 4B). At matched depths of 2 and 4 mm, the signal-to-background ratios for COE-BBT were ~3.7 and ~3.2, respectively, which are 2.2-fold and 2.4-fold higher than that for ICG (Fig. 4C). Similarly, compared to the commercial cell membrane cyanine dye 1,1′-dioctadecyl-3,3,3′,3′-tetramethylindotricarbocyanine iodide (DiR), COE-BBT@CAR-NK cells exhibited remarkable penetration depths in both 1% intralipid and chicken tissue (Fig. S5). When the chicken breast tissue thickness reached 8 mm, fluorescence from the COE-BBT@CAR-NK samples remained clearly detectable. In contrast, under the same imaging conditions, fluorescence from the DiR-labeled samples was barely detectable through 4 mm of chicken breast tissue (Fig. S5b). Furthermore, the minimal detection limit under in vitro conditions is ~20 labeled cells (Fig. 4D). These results demonstrate the high detectability of COE-BBT@CAR-NK cells and delineate an improved trade-off among cellular specificity, sensitivity, and penetration depth.

Subsequently, the in vivo imaging sensitivity of COE-BBT-labeled CAR-NK cells was also examined. To directly relate image contrast to the number of labeled cells in the field of view, COE-BBT@CAR-NK cells were injected intratumorally into flank LN229 tumors. The image contrast increased markedly relative to both the preinjection background and the unlabeled CAR-NK controls, indicating the capability of COE-BBT to label CAR-NK cells for NIR-II in vivo visualization (Fig. S6a and b). Following in vivo imaging, tumors were harvested, sectioned, and imaged by fluorescence microscopy to confirm successful delivery of labeled CAR-NK cells (Fig. S6c). Finally, we determined the in vivo detection limit by titrating the number of injected COE-BBT@CAR-NK cells (Fig. 4E). The NIR-II fluorescence of the labeled cells was observed around the injection site, and the integrated intensity was positively correlated with the number of cells administered (Fig. 4F). Notably, as few as ~50 labeled cells generated a discernible signal in vivo. These findings support the premise that COE-BBT labeling enables high-contrast imaging of CAR-NK cells within solid tumors, thereby establishing a technological foundation for subsequent in vivo tracking.

### In vivo tracking of COE-BBT@CAR-NK cell proliferation

**Fig. 5.**
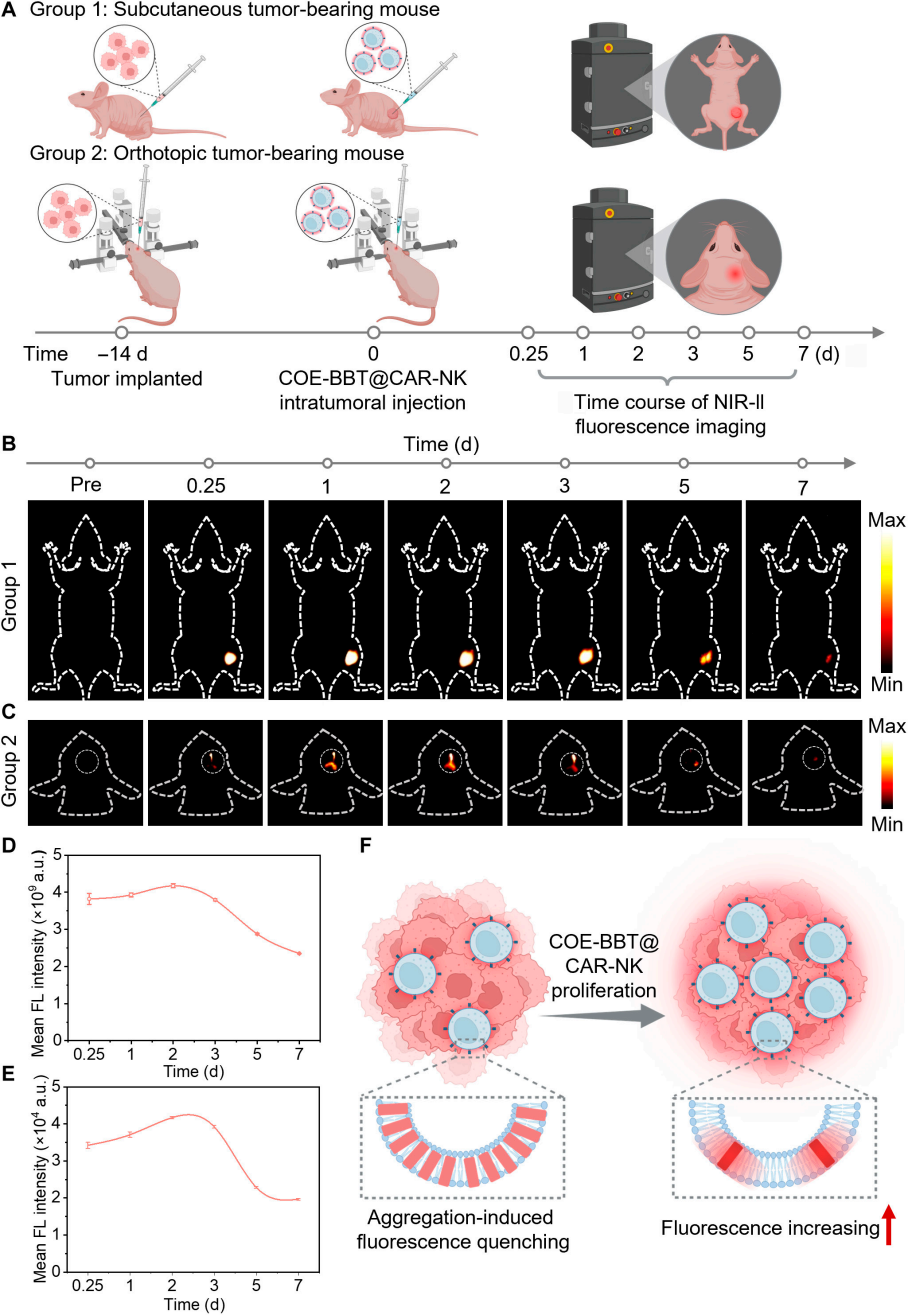
COE-BBT@CAR-NK cell in vivo tracking with second near-infrared (NIR-II) fluorescence (FL) imaging. (A) Schematic of experiment design. (B) NIR-II FL imaging (*λ*_exc_ = 808 nm) of subcutaneous glioma-bearing mice at various time points post-COE-BBT@CAR-NK cell intratumoral injection. (C) NIR-II FL imaging (*λ*_exc_ = 808 nm) of orthotopic glioma-bearing mice at various time points post-COE-BBT@CAR-NK cell intratumoral injection. Images captured utilizing a 1,000-nm long-pass filter, 500-ms exposure time. (D) FL intensity quantification of subcutaneous tumor post-COE-BBT@CAR-NK cell injection in panel (B). Data represent mean ± SD, *n* = 3. (E) FL intensity quantification of orthotopic tumor post-COE-BBT@CAR-NK cell injection in panel (C). Data represent mean ± SD, *n* = 3. (F) Schematic of aggregation-induced fluorescence quenching (ACQ)-enabled NIR-II signal amplification of COE-BBT during cell proliferation. The image was created using BioRender.com and is used with permission. COE-BBT, conjugated oligoelectrolytes-benzobisthiadiazole; CAR-NK cell, chimeric antigen receptor (CAR)-engineered natural killer cell.

To investigate the in vivo fate of COE-BBT-labeled CAR-NK cells, we then assessed the intratumoral persistence of COE-BBT@CAR-NK cells. Under in vitro observation, COE-BBT-labeled cells retained strong fluorescence after an 8-d incubation (Fig. S7), indicating stable probe retention and partitioning during cell division, thereby enabling longitudinal visualization. Building on this stability, COE-BBT@CAR-NK cells were intratumorally injected into subcutaneous or orthotopic LN229 glioma-bearing mice, and the NIR-II fluorescence signal was monitored over time (Fig. 5A). Consistent with the in vitro observations, NIR-II fluorescence dynamic imaging revealed a progressive increase in intensity during the first 2 d after intratumoral injection, followed by a reduction on day 3 (Fig. 5B and C). This early signal increase should be distinguished from the initial fluorescence turn-on that occurs when COE-BBT transfers from aqueous medium into the lipid bilayer. After membrane labeling has been established, COE-BBT remains membrane associated and undergoes a second fluorescence modulation during cell proliferation. Specifically, because the COE-BBT probe exhibits aggregation-induced fluorescence quenching at a relatively high local membrane density, redistribution of the probe during early cell division leads to greater intermolecular separation and reduced self-quenching, resulting in an initial increase in NIR-II fluorescence despite dilution of the membrane probe (Fig. 5F). The further reduction reflects the exhaustion of COE-BBT@CAR-NK cells in the complex tumor microenvironment. Consequently, this dequenching enables longitudinal in vivo tracking of cellular fate with COE-BBT labeling. Indeed, intratumoral NIR-II signals from orthotopically implanted COE-BBT@CAR-NK cells persisted over 7 d (Fig. 5D and E). Collectively, these results demonstrate that COE-BBT labeling enables long-term in vivo tracking of CAR-NK cells with a sufficient temporal window to monitor early expansion and subsequent dynamics within solid tumors.

### In vivo tracking of COE-BBT@CAR cell migration

In the previous section, we assessed the feasibility of in vivo tracking of COE-BBT@CAR-NK cell proliferation. Intratumoral administration is a common route in ACT for treating localized solid tumors, as it minimizes the accumulation of therapeutic cells in healthy organs and thereby reduces systemic toxicity [32]. However, recent studies have shown that NK cells can be rapidly exhausted after tumor entry due to the immunosuppressive microenvironment [33]. Alternatively, peritumoral injection places CAR cells in a less extreme niche, which may reduce primary exposure to the suppressive intratumoral cues [34]. Given that the directional migration of CAR cells toward tumors is closely correlated with their activation status and underpins cytotoxic efficacy [35], we evaluated the in vivo migration of COE-BBT@CAR-NK and CAR-T cells by peritumoral administration.

**Fig. 6.**
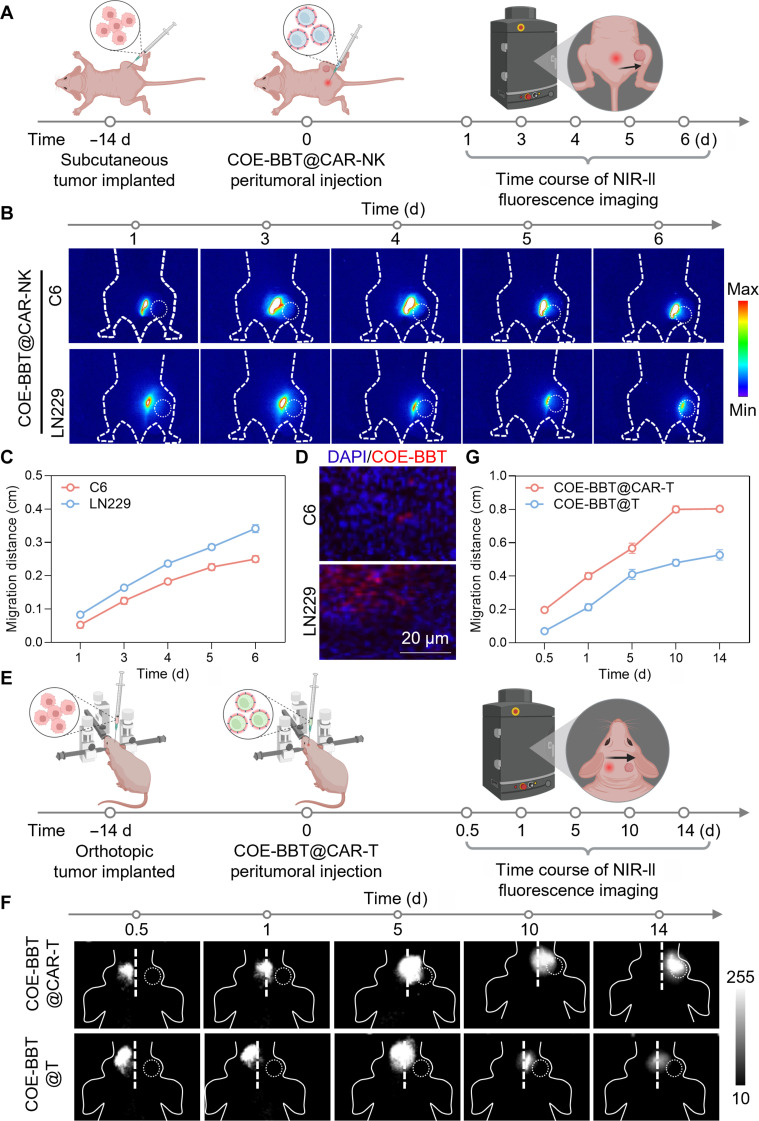
Characterization of COE-BBT@CAR-NK and CAR-T cell migration ability toward glioma. (A) Schematic of experiment design. The image was created using BioRender.com and is used with permission. (B) Second near-infrared (NIR-II) fluorescence (FL) imaging (*λ*_exc_ = 808 nm) of LN229 and C6 subcutaneous glioma-bearing mice at various time points postinjection with COE-BBT@CAR-NK cells at a distance of 0.5 cm. Images captured utilizing a 1,000-nm long-pass filter, 500-ms exposure time. (C) FL intensity quantification of the subcutaneous tumor from panel (B). Data represent mean ± SD, *n* = 3. (D) Confocal images of LN229 and C6 tumor sections (nuclei: blue) postinjected with COE-BBT@CAR-NK cells (red). Scale bar = 20 μm. (E) Schematic of experiment design. The image was created using BioRender.com and is used with permission. (F) NIR-II FL imaging (*λ*_exc_ = 808 nm) of LN229 orthotopic glioma-bearing mice at various time points postinjection with COE-BBT@CAR-T and COE-BBT@T cells at a distance of 1 cm. Images captured utilizing a 1,000-nm long-pass filter, 500-ms exposure time. (G) FL intensity quantification of the orthotopic tumor from panel (F). Data represent mean ± SD, *n* = 3. COE-BBT, conjugated oligoelectrolytes-benzobisthiadiazole; CAR-NK cell, chimeric antigen receptor (CAR)-engineered natural killer; CAR-T cell, CAR-engineered T cell.

Subcutaneous models were established using LN229 human glioma cells and compared with C6 rat glioma cells as controls. Fourteen days after tumor implantation, COE-BBT@CAR-NK cells were injected 0.5 cm from the center of LN229 or C6 gliomas. NIR-II imaging was then performed to monitor the migration of CAR-NK cells toward the tumor (Fig. 6A). The results indicate that COE-BBT@CAR-NK cells progressively migrated toward the tumor center, with a greater migration distance toward LN229 tumors than toward C6 tumors (Fig. 6B and C). The tumors were harvested postimaging, and fluorescence microscopy confirmed that COE-BBT@CAR-NK cell accumulation was markedly higher in LN229 sections than in C6 tumors (Fig. 6D), indicating preferential targeting of LN229 gliomas.

Subsequently, we evaluated the migration of COE-BBT@CAR-T and COE-BBT@T cells toward the tumor center in orthotopic LN229 glioma models (Fig. 6E). Both populations migrated toward the orthotopic tumor site, with COE-BBT@CAR-T cells traveling remarkably farther than COE-BBT@T cells (Fig. 6F and G). These results demonstrate that CAR engineering enhances the migration of immune cells toward LN229 tumors, confirming the improved tumor targeting conferred by CAR modification. The current findings support our hypothesis that COE-BBT labeling of immune cells enables tracking in deep tumors and dynamically captures their spatiotemporal migration within the tumor microenvironment.

### COE-BBT@CAR cell tumor accumulation across the blood–brain barrier

For the treatment of disseminated or surgically inaccessible tumors, CAR-expressing immune cells are typically administered intravenously [36]. However, systemic delivery of CAR-engineered cells to intracranial glioma can be constrained by inefficient trafficking across the blood–brain barrier (BBB), a protective structure formed by endothelial cells lining brain blood vessels [37–39]. Encouraged by evidence that COE-BBT@CAR-NK and T cells actively migrate toward tumor cells in a CAR-dependent manner, we further evaluated the tumor-targeting ability of COE-BBT@CAR-NK cells upon intravenous administration. The tumor-homing ability of COE-BBT@CAR-NK cells was first demonstrated in LN229 and C6 subcutaneous tumor-bearing mice. After receiving an intravenous administration of COE-BBT-labeled CAR-NK or NK cells, respectively, the biodistribution of COE-BBT@CAR-NK cells was quantified using NIR-II fluorescence imaging. Consistent with previous observation, both COE-BBT@CAR-NK and COE-BBT@NK cells accumulated at LN229 tumors with signals peaking at ~24 h, whereas signals for NK cells were barely observable in C6 tumors (Fig. S8). Compared with normal NK cells, which have modest glioma-targeting ability, CAR-NK cells showed higher tumor-region fluorescence and a longer persistence window.

**Fig. 7.**
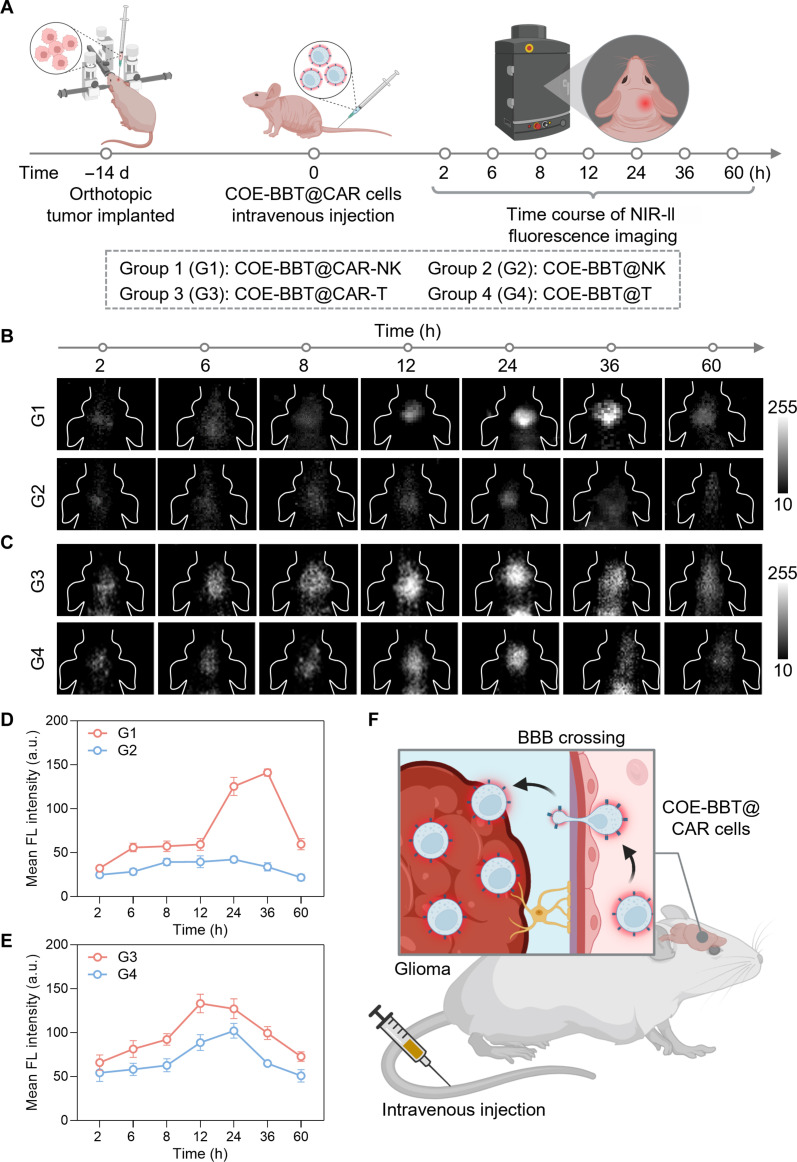
Characterization of the glioma-targeting ability of COE-BBT@CAR-NK/T cells using second near-infrared (NIR-II) fluorescence (FL) imaging. (A) Schematic of experiment design. (B) NIR-II FL imaging (*λ*_exc_ = 808 nm) of LN229 orthotopic glioma-bearing mice at various time points post-intravenous injection of COE-BBT@CAR-NK and COE-BBT@NK cells. (C) NIR-II FL imaging (*λ*_exc_ = 808 nm) of LN229 orthotopic glioma-bearing mice at various time points post-intravenous injection of COE-BBT@CAR-T and COE-BBT@T cells. Images captured utilizing a 1,000-nm long-pass filter, 500-ms exposure time. (D) FL intensity quantification of orthotopic tumor from panel (B). (E) FL intensity quantification of orthotopic tumor from panel (C). Data represent mean ± SD, *n* = 3. (F) Schematic of COE-BBT-labeled CAR-expressing cells tumor-targeted migration across the blood–brain barrier (BBB) post-intravenous injection. The image was created using BioRender.com and is used with permission. COE-BBT, conjugated oligoelectrolytes-benzobisthiadiazole; CAR-NK cell, chimeric antigen receptor (CAR)-engineered natural killer; CAR-T cell, CAR-engineered T cell.

To characterize the intracranial tumor targeting of COE-BBT-CAR-NK cells across the BBB, we subsequently performed experiments in an orthotopic LN229 model (Fig. 7A). Remarkably, both injected COE-BBT@CAR-NK and NK cells were found enriched in the brain, with peak intensities at 36 h postinjection (Fig. 7B), suggesting the tumor-targeting ability of NK cells that cross the BBB. Moreover, the fluorescence intensity of the COE-BBT@CAR-NK group increased ~4.2-fold in comparison to that of the COE-BBT@NK group (Fig. 7D), indicating the significant improvement of the tumor-targeting ability of NK cells upon CAR engineering. To examine the broad application of COE-BBT for cell labeling and further confirm the enhanced targeting conferred by CAR modification, the tumor-targeting behavior of COE-BBT@CAR-T cells was further evaluated in the orthotopic LN229 glioma model. Similarly, both COE-BBT@CAR-T and COE-BBT@T cells accumulated in the orthotopic gliomas, with peak signals at 12 and 24 h, respectively (Fig. 7C). Although the CAR modification in T cells leads to a marginal improvement (~1.5-fold) compared with that in NK cells (~4.2-fold), accelerated accumulation was observed in COE-BBT@CAR-T cells (Fig. 7E).

To further evaluate the systemic distribution of labeled cells after intravenous administration, ex vivo NIR-II fluorescence imaging of major organs (heart, liver, spleen, lung, kidneys, and brain) was performed. The fluorescence signals detected in the brain post-COE-BBT@CAR-NK injection provide further support for brain accumulation after intravenous delivery. Strong hepatic fluorescence also indicated that CAR-NK cells predominantly metabolize in the liver (Fig. S9). Taken together, the results above further demonstrate that COE-BBT labeling preserves the CAR-mediated glioma-targeting ability of NK and T cells. The COE-BBT probe enabled long-term in vivo tracking of CAR-modified immune cells, thereby allowing complete monitoring of BBB crossing, tumor accumulation, and clearance kinetics activities (Fig. 7F).

### Biosafety evaluation of COE-BBT and labeled CAR-NK cells

Lastly, we determined the biocompatibility of COE-BBT and COE-BBT@CAR-NK cells. An in vitro hemolysis assay was performed with COE-BBT at various concentrations. As a result, the hemolytic percentage of red blood cells at 64 μM was only 3.8%, indicating the favorable hemocompatibility of the designed probe (Fig. S10). Blood routine and biochemical indices were also measured to confirm the in vivo biosafety of COE-BBT@CAR-NK cells. Blood samples were collected from BALB/c mice on days 1, 4, and 7 post-intravenous injection of 5 × 10^6^ COE-BBT@CAR-NK cells. All 15 hematological parameters obtained from the blood routine test remained within normal ranges, exhibiting mild effects of COE-BBT@CAR-NK cells on immune response, oxygen transport capacity, and coagulation function (Fig. S11). Blood biochemical analysis indicated that throughout the monitoring period, 3 liver function markers (alanine aminotransferase, aspartate aminotransferase, and albumin), 2 renal function parameters (creatinine and urea), and 1 metabolic indicator (cholesterol) remained within normal ranges (Fig. S12), indicating that COE-BBT@CAR-NK cells induced no hepatic or renal impairment. These results confirmed the absence of acute toxicity and provided key evidence of the safety profile of COE-BBT@CAR-NK cells.

To evaluate the impact of COE-BBT@CAR-NK cells on vital organs, mice were sacrificed 24 h after COE-BBT@CAR-NK cell injection. Major organs, including the heart, liver, spleen, lungs, and brain, were harvested and stained with hematoxylin and eosin for histological examination. As shown in Fig. S13, no morphological alterations were observed in any of the examined tissues compared with untreated control groups. Together, these experimental data demonstrate that COE-BBT@CAR-NK cells exhibit favorable in vivo biocompatibility without eliciting significant toxicity. This provides crucial safety evidence supporting their further clinical translation, particularly for applications such as NIR-II fluorescence imaging.

## Conclusion

In summary, this study establishes COE-BBT as an ultrasensitive, background-free NIR-II probe for real-time tracking of ACT cells in glioma. By stably anchoring within lipid bilayers via synergistic electrostatic and hydrophobic interactions, COE-BBT enables high-efficiency membrane labeling across multiple tested cell types, including CAR-NK and CAR-T cells, without perturbing phenotypic profiles. In the present study, we primarily focused on CAR-NK cells as the representative ACT platform to evaluate the imaging performance and biological compatibility of COE-BBT in glioma models. Remarkably, the superior penetration depth and signal-to-background ratio performance enable ultrasensitive quantification of ~20 labeled cells in vitro and ~50 cells in vivo, supporting COE-BBT as a highly sensitive NIR-II probe for ACT cell tracking. In subcutaneous and orthotopic glioma models, COE-BBT, when combined with NIR-II imaging, provides longitudinal visualization following intratumoral, peritumoral, and intravenous administration, thereby resolving early expansion dynamics, persistence, directional migration, and tumor homing across the BBB. Biosafety evaluation further indicates favorable hemocompatibility and the absence of acute systemic toxicity. Although COE-BBT demonstrates broad membrane-labeling potential, further investigation is warranted to systematically characterize its labeling performance, cell-type-dependent effects, and broader applicability across diverse therapeutic applications. Collectively, this scalable, noninvasive platform enables quantitative, spatiotemporal dissection of CAR cell behavior in solid tumors, offering a practical route toward image-guided dose optimization and treatment monitoring in clinical ACT.

## Materials and Methods

### Materials

FITC anti-human CD3 and APC anti-human CD16 were purchased from BioLegend (USA); PE anti-human CD56 (neural cell adhesion molecule) and V5 Tag Monoclonal Antibody manganese chloride were purchased from Thermo (USA). The cell culture components were all purchased from Gibco (USA) and iCell Bioscience Inc. (Shanghai, China). NK cells of human malignant non-Hodgkin’s lymphoma (NK-92 MI; Cat. No.: iCell-h331) were obtained from iCell Bioscience Inc. (Shanghai, China), rat glioma (C6; Cat. No.: CCL-107) and mouse monocyte-macrophage leukemia RAW 264.7 cells (Cat. No.: TIB-71) were obtained from American Type Culture Collection (USA), and human glioblastoma cells (LN229; Cat. No.: CTCC-400-0015) were obtained from MeisenCTCC (Zhejiang, China).

### COE-BBT fluorescence probe synthesis and characterization

The COE-BBT probe was synthesized and characterized as previously described [29]. Briefly, BBT-Br (196 mg, 0.110 mmol) was dissolved in chloroform (20 ml) in a single-neck round-bottom flask. A solution of trimethylamine in tetrahydrofuran (2 ml) was then added, and the mixture was stirred at 55 °C for 16 h. Upon completion, the pale supernatant was decanted, and the precipitated crude product adhering to the flask bottom was gently rinsed with chloroform 5 times. Subsequently, trimethylamine in methanol (2 ml) was added, and the mixture was stirred at 55 °C for 16 h. Solvents were then removed in a vortex vessel, and the residue was dried under vacuum to afford the COE-BBT fluorescence probe.

### Cell culture

C6, LN229, and RAW 264.7 cells were cultured in Dulbecco’s modified Eagle medium supplemented with 10% fetal bovine serum and 1% penicillin/streptomycin. NK-92MI and CAR-NK cells were cultured in minimum essential medium α media supplemented with 12.5% fetal bovine serum, 12.5% horse serum, 0.1% β-mercaptoethanol, 0.2 mM inositol, 0.02 mM folic acid, and IL-2 (10 ng/ml). CAR-T cells were maintained in ImmunoCult-XF T Cell Expansion Medium supplemented with IL-2 (10 ng/ml). Cells were maintained at 37 °C in 5% CO_2_.

### MD simulation investigation

Systematic MD simulations of the complex were performed using the ff14SB force field in the Amber2020 package under the isothermal–isobaric (NPT) ensemble with periodic boundary conditions. The Amber force field (ff14SB) and TIP3P water model were employed for all simulations. Prior to the MD simulations, a 2-step energy minimization procedure was conducted to eliminate unfavorable atomic contacts in the system: specifically, 5,000 steps of steepest descent followed by 5,000 steps of conjugate gradient were implemented for the restrained and unrestrained systems, respectively. The MD simulations were divided into 2 consecutive phases: in the first phase (0 to 5 ns), the solute was restrained, and the system was slowly heated up to 300 K; in the second phase (5 to 500 ns), unrestrained simulations were performed at a constant temperature, with the SHAKE algorithm applied to constrain all hydrogen-containing atoms. Other key simulation parameters were set as follows: the nonbonded cutoff radius was 1 nm, the integration time step was 2 fs, and the conformational-sampling interval was 10 ps. A total of 5 × 10^4^ conformational snapshots were captured and monitored using VMD and subsequently used for statistical analysis. Furthermore, constant-velocity steered MD simulations, in which the stretching velocity of the target atoms or molecules was held constant to drive their motion along a specific direction, were used to elucidate the relationship between the structures of staining agents and phospholipid bilayers.

### Isolation of murine peripheral blood neutrophils

Neutrophils were isolated from peripheral blood using a mouse neutrophil isolation kit. Peripheral blood was collected from healthy C57BL/C mice via the retro-orbital sinus and transferred into blood collection tubes preloaded with TBDTM anticoagulant. Separation Solution 1 (3 ml) was added to a centrifuge tube, followed by 3 ml of 80% Separation Solution 1 to form a density gradient interface. Anticoagulated blood was then mixed 1:1 with erythrocyte sedimentation solution and carefully layered onto the separation media, followed by a 20-min centrifugation at 800*g*. The lower neutrophil layer was collected, mixed with washing buffer (3 ml), and centrifuged for 10 min at 400*g*. The pellet was then collected and treated with red blood cell lysis buffer (1 ml) at room temperature for 3 min, and neutrophils were pelleted at 500*g* for 5 min.

### Vector components and sequences

#### 14G2A anti-GD2 scFv

D​VVM​TQT​PLS​LPV​SLG​DQA​SIS​CRS​SQS​LVH​RNG​NTY​LHW​YLQ​KPG​QSP​KLL​IHK​VSN​RFS​GVP​DRF​SGS​GSG​TDF​TLK​ISR​VEA​EDL​GVY​FCS​QST​HVP​PLT​FGA​GTK​LEL​KRG​GGG​SGG​GGS​GGG​GSE​VQL​LQS​GPE​LEK​PGA​SVM​ISC​KAS​GSS​FTG​YNM​NWV​RQN​IGK​SLE​WIG​AID​PYY​G​GTS​YNQ​KFK​GRA​TLT​VDK​SSS​TAY​MHL​KSL​TSE​DSA​VYY​CVS​GMK​YWGQGTSVTVSS

#### V5 tag

GKPIPNPLLGLDST

#### CD8 hinge

F​VPV​FLP​AKP​TTT​PAP​RPP​TPA​PTI​ASQ​PLS​LRP​EAC​RPA​AGG​AVH​TRG​LDF​ACD​IYI​WAP​LAG​TCGVLLLSLVITLYCNHRN

#### 4-1BB

K​RGR​KKL​LYI​FKQ​PFM​RPV​QTT​QEE​DGC​SCR​FPE​EEEGGCEL

#### CD3z

R​VKF​SRS​ADA​PAY​QQG​QNQ​LYN​ELN​LGR​REE​YDV​LDK​RRG​RDP​EMG​GKP​RRK​NPQ​EGL​YNE​LQK​DKM​AEA​YSE​IGMKGERRRGKG​HDGLYQGLSTATKDTYDALHMQALPPR

### Lentiviral vectors construction and cell transfection

The GD2 CAR nLuc vector was constructed using the scFv region of the GD2 antibody (clone 14G2A) and a typical second-generation 4-1BB CD3 zeta CAR sequence, linked to a V5 tag and a CD8 hinge. The nano-luciferase coding DNA sequence was derived from AHH41352.1 and primed with another independent promoter. The whole sequence is cloned into a pCDH lentiviral backbone (System Biosciences), The viral packaging helper plasmids were psPAX2 (Addgene, 12260) and pMD2.G (Addgene, 12259).

The GD2 CAR nLuc lentiviral vector and the helper plasmids were cotransfected into HEK 293T cells at a ratio of 10:7:3 using PEI 40K (Polysciences, no. 24765-1). Eight hours posttransfection, the culture media were replaced, and 48 h posttransfection, the media were harvested and centrifuged for 5 min at 400*g* to remove cell debris. The supernatant was sterilized with a 0.45-mm filter. The NK-92 MI cells were then infected with the supernatant, diluted 1:1 with NK-92 MI culture media. Two days postinfection, cells were collected and stained with V5 tag FACS antibody (Thermo Fisher, no. 12-6796-42), the transduction efficiency was analyzed, and the positive cells were isolated using a flow cytometer sorter. The GD2 CAR-T cells were generated and characterized in Zhang’s lab as previously described [40].

### In vitro cytotoxicity assay

GD2 CAR-NK cells show specific cytotoxicity against LN229 cells. LN229 cells labeled with CellTrace Far Red dye (Thermo Fisher, C34564) were co-cultured with CAR-NK cells (E:T = 5:1) in Incucyte (Sartorius Incucyte). At 18 h, phase and red fluorescence were recorded and overlaid. The cell lysis ratio at the indicated time points was measured by Incucyte analysis.

### Cell labeling with the COE-BBT fluorescence probe

To determine the optimal concentration of COE-BBT, 1 × 10^6^ cells/ml of CAR-NK cells were collected, resuspended in culture media, and incubated with COE-BBT at 2, 4, 8, 10, or 20 μM for 12 h. Cells were then pelleted at 500*g* for 3 min, washed twice with PBS, and resuspended in 96-well plates (1 × 10^4^ cells per well). Fluorescence intensity was quantified using an NIR-II imaging system (NIRvana 640, Teledyne Princeton Instruments) to determine the optimal labeling concentration. For time-course optimization, COE-BBT at the determined optimal concentration (10 μM) was incubated with CAR-NK cells (1 × 10^6^ cells/ml) for 1, 2, 4, 8, 12, or 24 h. Cells were collected, and fluorescence intensity was quantified as described above.

For the labeling of suspension cells, CAR-T cells and neutrophils were collected and resuspended in media at 1 × 10^6^ cells/ml. COE-BBT (10 μM) was added, and cells were incubated at 37 °C for 12 h. Cells were then pelleted at 500*g* for 3 min and washed twice with PBS. For adherent cells, 1 × 10^6^ cells/ml RAW 264.7 and LN229 cells were seeded and incubated with COE-BBT (10 μM) at 37 °C for 12 h. Cells were then trypsinized, pelleted at 500*g* for 3 min, and washed twice with PBS. The pellet was resuspended in the culture medium for subsequent in vitro or in vivo studies. Fluorescence intensity was quantified using an NIR-II imaging system using unlabeled cells as a control, and labeling efficiency was assessed via flow cytometry analysis (excitation 405 nm and emission 525 nm).

### Characterization of COE-BBT-labeled cells

The viability of COE-BBT-labeled CAR-NK cells was determined using Cell Counting Kit-8 (CCK-8, MedChemExpress); 1 × 10^6^ cells per well were seeded into 96-well plates and incubated with COE-BBT. For each concentration (0, 1, 2, 4, 8, 16, and 32 μM), 3 replicate wells were included. After 12 h of incubation at 37 °C, 10 μl of the CCK-8 reagent was added to each well and incubated for 1 h at 37 °C. The absorbance of the tested wells was measured at 450 nm using a microplate reader to calculate cell viability.

The proliferation of COE-BBT-labeled CAR-NK cells was calculated via cell counting. Briefly, 1 × 10^6^ cells/ml of CAR-NK cells, with and without COE-BBT labeling, were resuspended in culture media, and the total cell number was monitored on days 0, 1, 3, 5, and 7 using an automated cell counter upon trypan blue staining.

The immunophenotypic profile of the COE-BBT-labeled CAR-NK cells was characterized upon key surface marker staining; 5 × 10^5^ cells/ml of CAR-NK cells, with and without COE-BBT labeling, were incubated with 2.5 μl of anti-CD3 antibody together with 2.5 μl of anti-CD16 or anti-CD56 antibody at 37 °C for 15 min. Cells were then pelleted, washed twice with PBS, and resuspended in 500 μl of ice-cold PBS. The immunophenotypic profile was characterized by flow cytometry.

The apoptosis of CAR-NK cells with and without COE-BBT labeling was determined using the Annexin V-FITC Apoptosis Detection Kit (Beyotime, China); 5 × 10^5^ cells from each condition were collected 3 d postlabeling and washed twice with PBS. Then, the cells were suspended in 100 μl of PBS containing 5 μl of annexin V–FITC and 10 μl of PI, mixed gently, and incubated for 15 min at room temperature. Then, 400 μl of PBS was added, and the samples were kept on ice. The fluorescence of FITC and PI was detected through flow cytometry (Beckman CytoFLEX, Ex 405 nm, Em 525 nm).

To assess cytokine secretion, 5 × 10^5^ CAR-NK cells, with and without COE-BBT labeling, were seeded into 24-well plates and incubated for 24 h. The culture media were then collected, and the concentrations of IFN-γ, TNF-α, and IL-2 were quantified using the corresponding ELISA kits (BioLegend, no. 505807; MedChemExpress, no. HY-P80914; MedChemExpress, no. HY-P7077) according to the manufacturers’ protocols.

### Characterization of fluorescence penetration depth

For penetration-depth analysis, CAR-NK cells labeled with COE-BBT, DiR, or ICG were loaded into glass capillary tubes and placed in a 10-cm culture dish. The tubes were then covered with 1% intralipid of a defined depth (0 to 9 mm). For the penetration assay with chicken breast tissue, CAR-NK cells labeled with COE-BBT or DiR were placed into a 96-well plate and covered with fresh chicken breast tissue of a defined thickness (0, 2, 4, 6, and 8 mm). The NIR-II fluorescence was acquired under identical imaging settings (*λ*_exc_ = 808 nm, 1,000-nm long-pass filter, 1,200-ms exposure time), and the resulting images were quantified using Fiji ImageJ.

### Animal model

Female NSG mice weighing 18 to 22 g were obtained from Guangdong Yaokang Biotechnology Co., Ltd. (China). Female BALB/c mice weighing 18 to 22 g were acquired from Guangdong Charles River Laboratory Animal Technologies Co., Ltd. (China). All animal experiments were approved by the Animal Care and Use Committee of Shenzhen Institutes of Advanced Technology, Chinese Academy of Sciences (China) (SIAT-IACUC-220519-YGS-SZH-A2148).

### Establishment of the orthotopic glioma mouse model

An orthotopic glioma model was established using luciferase-expressing LN229 cells. The mice were anesthetized with isoflurane gas and secured in a stereotaxic frame. Following shave, skin disinfection, and midline scalp incision, a microsyringe was positioned at the predefined stereotaxic coordinates (3.0 mm from bregma, 2.0 mm lateral to the sagittal suture, and 3.5 mm in depth), and 5 × 10^5^ LN229 cells suspended in 5 μl of serum-free medium were slowly injected over 5 min. Tumor growth was subsequently monitored through bioluminescence imaging.

### In vivo imaging of COE-BBT-labeled CAR-NK cells

To characterize the in vivo fluorescence penetration depth, 50, 100, 200, 500, and 1,000 labeled cells were resuspended in 50 μl of PBS, and fluorescence intensity was measured with the NIR-II imaging system. Subsequently, aliquots containing the indicated numbers of labeled cells were injected intratumorally into tumor-bearing nude mice, and in vivo NIR-II fluorescence was recorded.

To monitor CAR-NK cell proliferation in vivo, the cells were prelabeled with COE-BBT as described above. Labeled cells (5 × 10^5^ cells) were injected into the intracranial orthotopic or subcutaneous glioma lesions of the mice. In vivo NIR-II imaging was performed at 0.25, 1, 2, 3, 5, and 7 d.

### Migration of CAR-NK and CAR-T cells targeting glioma tumors

Mice bearing subcutaneous LN229 or C6 gliomas with comparable volumes were selected and randomly assigned into different groups (*n* = 3 per group). The mice were injected with 1 × 10^6^ COE-BBT-labeled CAR-NK cells once adjacent to the respective subcutaneous tumor, and NIR-II imaging was performed at 1, 3, 4, 5, and 6 d to track cell migration toward the tumor. Migration was quantified as the distance between the fluorescence signal and the tumor center. For intracranial models, mice with orthotopic LN229 glioma were randomized into 2 groups (*n* = 3 per group) and received an intracerebroventricular injection of 1 × 10^6^ COE-BBT-labeled T or CAR-T cells, respectively. NIR-II imaging was performed at 0.5, 1, 3, 5, 7, 10, and 14 d postinjection to evaluate intracranial migration.

### Active targeting of glioma tumor by CAR-NK and CAR-T cells

LN229 glioma-bearing mice (subcutaneous or orthotopic sites) with comparable tumor volumes were selected and randomly assigned into different groups (*n* = 3 per group). The mice were injected via the tail vein with 5 × 10^5^ of COE-BBT-labeled CAR-NK or CAR-T cells, with nonengineered NK or T cells as the corresponding controls. In vivo fluorescence signals were acquired and analyzed using an NIR-II imaging system at 2, 6, 8, 12, 24, 36, and 60 h postinjection.

To evaluate the ex vivo biodistribution, glioma-bearing mice were sacrificed 24 h post-intravenous injection of COE-BBT-labeled CAR-NK and NK cells. Major organs, including the heart, liver, spleen, lung, kidneys, and brain, were harvested and imaged using an NIR-II imaging system.

### Hemolysis assay

Whole blood (2 ml) from healthy BALB/c mice was collected and mixed with 10 ml of PBS and then centrifuged at 1,000 rpm for 5 min to pellet erythrocytes. Harvested erythrocytes were washed 3 times with PBS to remove plasma components and then diluted with 0.9% NaCl solution to a final concentration of 2% (v/v). COE-BBT was diluted in PBS to obtain a series of solutions at 2, 4, 8, 16, 32, 64, and 128 μM. Distilled water was used as the positive control to achieve complete hemolysis. For each condition, 500 μl of the 2% erythrocyte suspension was mixed with 500 μl of the COE-BBT solutions at corresponding concentrations and incubated at 37 °C and 200 rpm on a shaker for an hour. After incubation, samples were centrifuged at 5,000 rpm for 5 min, and 150 μl of supernatant was transferred to a 96-well plate. The absorbance at 540 nm was measured on a microplate reader to evaluate the hemolytic rate of COE-BBT.

### In vivo safety evaluation

To comprehensively assess the biosafety of COE-BBT-labeled CAR-NK cells, healthy BALB/c mice were randomly assigned to 4 groups (*n* = 5 per group). The control group received PBS injection (100 μl). The remaining groups received an injection of 5 × 10^6^ COE-BBT-labeled CAR-NK cells, and samples were collected on days 1, 4, and 7. Blood samples were collected, and routine hematological and serum biochemical examinations were performed to determine potential hematologic or hepatic/renal toxicity induced by COE-BBT or CAR-NK cells. Major organs (heart, liver, spleen, lungs, kidneys, and brain) were subsequently harvested from each sacrificed mouse and subjected to hematoxylin–eosin staining for histopathological analysis.

## Ethical Approval

All animal experiments were approved by the Animal Care and Use Committee of Shenzhen Institutes of Advanced Technology, Chinese Academy of Sciences (China) (SIAT-IACUC-220519-YGS-SZH-A2148).

## Data Availability

All essential data are provided within the article and its supplementary materials, and further information is available from the corresponding authors on request.

## References

[1] Peng L, Sferruzza G, Yang L, Zhou L, Chen S. CAR-T and CAR-NK as cellular cancer immunotherapy for solid tumors. Cell Mol Immunol. 2024;21:1089–1108. 39134804 10.1038/s41423-024-01207-0 PMC11442786

[2] Baulu E, Gardet C, Chuvin N, Depil S. TCR-engineered T cell therapy in solid tumors: State of the art and perspectives. Sci Adv. 2023;9(7):eadf3700. 36791198 10.1126/sciadv.adf3700 PMC9931212

[3] Lai M, Shao W, Mao J, Ye Q. Revolution in cell therapy: In vivo chimeric-antigen-receptor-T-cell therapy breakthroughs and promises for the future. Research. 2025;8:0917. 41079670 10.34133/research.0917 PMC12509061

[4] Zhang P, Zhang G, Wan X. Challenges and new technologies in adoptive cell therapy. J Hematol Oncol. 2023;16:97. 37596653 10.1186/s13045-023-01492-8 PMC10439661

[5] Maalej KM, Merhi M, Inchakalody VP, Mestiri S, Alam M, Maccalli C, Cherif H, Uddin S, Steinhoff M, Marincola FM, et al. CAR-cell therapy in the era of solid tumor treatment: Current challenges and emerging therapeutic advances. Mol Cancer. 2023;22:20. 36717905 10.1186/s12943-023-01723-z PMC9885707

[6] Li Q, Hu D, Gao D, Gao G, Zhang C, Sheng Z. Optical imaging of in vivo adoptive T-cell therapy: State of the art and challenges. iRADIOLOGY. 2023;1:225–235.

[7] Morath V, Fritschle K, Warmuth L, Anneser M, Dötsch S, Živanić M, Krumwiede L, Bösl P, Bozoglu T, Robu S, et al. PET-based tracking of CAR T cells and viral gene transfer using a cell surface reporter that binds to lanthanide complexes. Nat Biomed Eng. 2025;9(11):1886–1906. 40514433 10.1038/s41551-025-01415-7 PMC12623248

[8] Raj SS, Fei T, Fried S, Ip A, Fein JA, Leslie LA, Tomas AA, Liethner D, Peled JU, Corona M, et al. An inflammatory biomarker signature of response to CAR-T cell therapy in non-Hodgkin lymphoma. Nat Med. 2025;31:1183–1194. 40169864 10.1038/s41591-025-03532-x PMC12003198

[9] Fröse J, Rowley J, Farid AS, Rakhshandehroo T, Leclerc P, Mak H, Allen H, Moravej H, Munaretto L, Millan-Barea L, et al. Development of an antigen-based approach to noninvasively image CAR T cells in real time and as a predictive tool. Sci Adv. 2024;10(38):eadn3816. 39292778 10.1126/sciadv.adn3816 PMC11409975

[10] Kurtz K, Eibler L, Dacek MM, Carter LM, Veach DR, Lovibond S, Reynaud E, Qureshy S, McDevitt MR, Bourne C, et al. Engineering CAR-T cells for radiohapten capture in imaging and radioimmunotherapy applications. Theranostics. 2023;13(15):5469–5482. 37908719 10.7150/thno.87489 PMC10614694

[11] Pham TT, Chenoweth A, Patel N, Banu A, Osborn G, Blower PJ, Karagiannis SN, Ma MT. In vivo PET imaging of ^89^Zr-labeled natural killer cells and the modulating effects of a therapeutic antibody. J Nucl Med. 2024;65(7):1035–1042. 38844362 10.2967/jnumed.124.267876 PMC11218727

[12] Kubelick KP, Kim J, Kim M, Huang X, Wang C, Song S, Xia Y, Emelianov SY. *In vivo* ultrasound and photoacoustic imaging of nanoparticle-engineered T cells and post-treatment assessment to guide adoptive cell immunotherapy. ACS Nano. 2025;19:6079–6094. 39908484 10.1021/acsnano.4c12929 PMC11841050

[13] Chen H, Machado A, An D, Becharef S, Autret G, Ayollo D, Razafindrakoto S, Nizard P, Carn F, Luo Y, et al. In vivo monitoring and magnetically-enhanced delivery of CAR T-cells to solid tumor. Adv Funct Mater. 2025;35(5):2414368.

[14] Hunger J, Schregel K, Boztepe B, Agardy DA, Turco V, Karimian-Jazi K, Weidenfeld I, Streibel Y, Fischer M, Strum V, et al. *In vivo* nanoparticle-based T cell imaging can predict therapy response towards adoptive T cell therapy in experimental glioma. Theranostics. 2023;13(15):5170–5182. 37908732 10.7150/thno.87248 PMC10614679

[15] Jiang Y, Ren T, Zhao S, Baghdasaryan A, Zhang X, Chen X, Wang F, Dai H. In vivo imaging of the immune system. Nat Rev Bioeng. 2026;4:454–474.

[16] Hu D, Zha M, Zheng H, Gao D, Sheng Z. Recent advances in indocyanine green-based probes for second near-infrared fluorescence imaging and therapy. Research. 2025;8:0583. 39830366 10.34133/research.0583 PMC11739436

[17] Feng Z, Li Y, Chen S, Li J, Wu T, Ying Y, Zheng J, Zhang Y, Zhang J, Fan X, et al. Engineered NIR-II fluorophores with ultralong-distance molecular packing for high-contrast deep lesion identification. Nat Commun. 2023;14:5017. 37596326 10.1038/s41467-023-40728-6 PMC10439134

[18] Chen Z, Huang L, Gao D, Bao Z, Hu D, Zheng W, Chen J, Liao J, Zheng H, Sheng Z. High spatiotemporal near-infrared II fluorescence lifetime imaging for quantitative detection of clinical tumor margins. Adv Sci. 2025;12(5): Article e2411272. 10.1002/advs.202411272 PMC11791973 39652447

[19] Chen Q-Y, Zhong Q, Liu Z-Y, Li P, Lin G-T, Zheng Q-L, Wang J-B, Lin J-X, Lu J, Cao L-L, et al. Indocyanine green fluorescence imaging-guided versus conventional laparoscopic lymphadenectomy for gastric cancer: Long-term outcomes of a phase 3 randomised clinical trial. Nat Commun. 2023;14:7413. 37973806 10.1038/s41467-023-42712-6 PMC10654517

[20] Ashmore-Harris C, Iafrate M, Saleem A, Fruhwirth GO. Non-invasive reporter gene imaging of cell therapies, including T cells and stem cells. Mol Ther. 2020;28(6):1392–1416. 32243834 10.1016/j.ymthe.2020.03.016 PMC7264441

[21] He J, Zhang C, Liang C, Xue W, Li Y, Dai L, Liu C, Zhuang W-R, Ma X, Cheng R, et al. Potentiating immunotherapy in “immune-cold” solid tumors through orchestrating T cell immunity via tumor-specific genetic engineering. Cell Rep Med. 2025;6(12): Article 102510. 41406950 10.1016/j.xcrm.2025.102510 PMC12765953

[22] Li H, Yang X, Wang Z, She W, Liu Y, Huang L, Jiang P. A near-infrared-II fluorescent nanocatalyst for enhanced CAR T cell therapy against solid tumor by immune reprogramming. ACS Nano. 2023;17(12):11749–11763. 37319120 10.1021/acsnano.3c02592

[23] Liao N, Su L, Zheng Y, Zhao B, Wu M, Zhang D, Yang H, Liu X, Song J. In vivo tracking of cell viability for adoptive natural killer cell-based immunotherapy by ratiometric NIR-II fluorescence imaging. Angew Chem Int Ed Engl. 2021;60:20888–20896. 34268865 10.1002/anie.202106730

[24] Sun Y, Li R, Cai Y, Liu Y, Wang P, Wu M, Zhang X, Liao N, Zhang C, Zheng A, et al. Two-plex *in vivo* molecular imaging in the second near-infrared window for immunotherapeutic response. Theranostics. 2025;15:4481–4494. 40225584 10.7150/thno.108880 PMC11984393

[25] Pfister F, Carnell LR, Löffler L, Boosz P, Schaft N, Dörrie J, Stein R, Lenz M, Speicker E, Huber CM, et al. Loading of CAR-T cells with magnetic nanoparticles for controlled targeting suppresses inflammatory cytokine release and switches tumor cell death mechanism. MedComm. 2020;2025(6): Article e70039. 10.1002/mco2.70039 PMC11702464 39764559

[26] Garner LE, Park J, Dyar SM, Chworos A, Sumner JJ, Bazan GC. Modification of the optoelectronic properties of membranes via insertion of amphiphilic phenylenevinylene oligoelectrolytes. J Am Chem Soc. 2010;132:10042–10052. 20608655 10.1021/ja1016156

[27] Zhou C, Chia GWN, Ho JCS, Moreland AS, Seviour T, Liedberg B, Parikh AN, Kjelleberg S, Hinks J, Bazan GC. A chain-elongated oligophenylenevinylene electrolyte increases microbial membrane stability. Adv Mater. 2019;31(18): Article e1808021. 30908801 10.1002/adma.201808021

[28] Zhou C, Ho JCS, Chia GWN, Moreland AS, Ruan L, Liedberg B, Kjelleberg S, Hinks J, Bazan GC. Gram-typing using conjugated oligoelectrolytes. Adv Funct Mater. 2020;30(42):2004068.

[29] Zhou C, Li Z, Zhu Z, Chia GWN, Mikhailovsky A, Vázquez RJ, Chan SJ, Li K, Liu B, Bazan GC. Conjugated oligoelectrolytes for long-term tumor tracking with incremental NIR-II emission. Adv Mater. 2022;34(20):2201989. 10.1002/adma.202201989 35306702

[30] Zhou C, Cox-Vázquez SJ, Chia GWN, Vázquez RJ, Lai HY, Chan SJW, Limongyut J, Bazan GC. Water-soluble extracellular vesicle probes based on conjugated oligoelectrolytes. Sci Adv. 2023;9:eade2996. 36630497 10.1126/sciadv.ade2996 PMC9833659

[31] Liu P, Chen Y, Yao D, Jia J, Meng Y, Zhou P, Gao X, Xie Y, Yao L, Li S, et al. Host–guest antimicrobial based on conjugated oligoelectrolyte and cyclodextrin. Angew Chem Int Ed Engl. 2025;64(30): Article e202504581. 40386884 10.1002/anie.202504581

[32] Brown CE, Hibbard JC, Alizadeh D, Blanchard MS, Natri HM, Wang D, Ostberg JR, Aguilar B, Wagner JR, Paul JA, et al. Locoregional delivery of IL-13Rα2-targeting CAR-T cells in recurrent high-grade glioma: A phase 1 trial. Nat Med. 2024;30(4):1001–1012. 38454126 10.1038/s41591-024-02875-1 PMC11031404

[33] Dean I, Lee CYC, Tuong ZK, Li Z, Tibbitt CA, Willis C, Gaspal F, Kennedy BC, Matei-Rascu V, Fiancette R, et al. Rapid functional impairment of natural killer cells following tumor entry limits anti-tumor immunity. Nat Commun. 2024;15:683. 38267402 10.1038/s41467-024-44789-z PMC10808449

[34] Zhang S, Regan K, Najera J, Grinstaff MW, Datta M, Nia HT. The peritumor microenvironment: Physics and immunity. Trends Cancer. 2023;9(8):609–623. 37156677 10.1016/j.trecan.2023.04.004 PMC10523902

[35] Song EZ, Timpanaro A, Meechan M, Elena-Sanchez L, Li LZ, Jamet S, Lau DS, Winter LI, Dun MD, Foster JB, et al. Engineered CXCR3-A expression enhances B7-H3-targeting CAR T cell migration and efficacy against diffuse intrinsic pontine glioma. Nat Commun. 2025;16:9914. 41219209 10.1038/s41467-025-64861-6 PMC12606338

[36] Dorff TB, Blanchard MS, Adkins LN, Luebbert L, Leggett N, Shishido SN, Macias A, Del Real MM, Dhapola G, Egelston C, et al. PSCA-CAR T cell therapy in metastatic castration-resistant prostate cancer: A phase 1 trial. Nat Med. 2024;30:1636–1644. 38867077 10.1038/s41591-024-02979-8 PMC11186768

[37] Wu D, Chen Q, Chen X, Han F, Chen Z, Wang Y. The blood–brain barrier: Structure, regulation, and drug delivery. Signal Transduct Target Ther. 2023;8:217. 37231000 10.1038/s41392-023-01481-w PMC10212980

[38] Ijaz M, Tan Q, Yan Y, Zhang D, Chen Q, Zhang Y, Tu Y, Guo B. Overcoming barriers in glioblastoma: The potential of CAR T cell immunotherapy. Theranostics. 2025;15(14):7090–7126. 40585996 10.7150/thno.114257 PMC12204080

[39] Yuan S, Hu D, Gao D, Butch CJ, Wang Y, Zheng H, Sheng Z. Recent advances of engineering cell membranes for nanomedicine delivery across the blood–brain barrier. J Nanobiotechnol. 2025;23:493. 10.1186/s12951-025-03572-y PMC12235962 40624508

[40] Zhu N, Chen S, Jin Y, Wang M, Fang L, Xue L, Hua D, Zhang Z, Jia M, Hao M, et al. Enhancing glioblastoma immunotherapy with integrated chimeric antigen receptor T cells through the re-education of tumor-associated microglia and macrophages. ACS Nano. 2024;18(17):11165–11182. 38626338 10.1021/acsnano.4c00050

