# Supplementary material for: Ultrasensitive In Vivo Imaging of Adoptive Immune Cell Distribution and Expansion Using Second Near-Infrared Conjugated Oligoelectrolyte Probes
Source: Research (Wash D C). 2026 Jun 26;9:1342. doi: 10.34133/research.1342 (PMC13305027; doi:10.34133/research.1342)
Supplement: Supplementary 1 — Figs. S1 to S13 [file research.1342.f1.docx]

SUPPLEMENTARY MATERIALS

Title

Ultrasensitive In Vivo Imaging of Adoptive Immune Cell Distribution and Expansion Using NIR-II Conjugated Oligoelectrolyte Probes

**Authors**

Shengnan Yuan^1,2†^, Qingshuang Li^1,2†^, Xi Kang^3†^, Yingying Meng^4^, Pengke Liu^4^, Pengfei Zhang^1,2^, Jin Zhang^1,2^, Dehong Hu^1,2^, Duyang Gao^1,2^, Caoyun Ju^5^, Xiuqi Li^5^, Can Zhang^5^, Hairong Zheng^1,2^, Nuernisha Alifu^6*^, Cheng Zhou^4*^, Zonghai Sheng^1,2*^

^1^Research Center for Advanced Detection Materials and Medical Imaging Devices, Institute of Biomedical and Health Engineering, Shenzhen Institute of Advanced Technology, Chinese Academy of Sciences, Shenzhen 518055, P. R. China.

^2^State Key Laboratory of Biomedical Imaging Science and System, Shenzhen 518055, P. R. China.

^3^Shenzhen Synthetica Pioneering Co. Ltd, 501 National Industrial Innovation Center for Bio-manufacturing, Shenzhen 518000, PR China.

^4^Institute of Polymer Optoelectronic Materials and Devices, Guangdong Basic Research Center of Excellence for Energy & Information Polymer Materials, State Key Laboratory of Luminescent Materials and Devices, School of Materials Science and Engineering, South China University of Technology, Guangzhou 510640, P. R. China

^5^State Key Laboratory of Natural Medicines and Jiangsu Key Laboratory of Drug Discovery for Metabolic Diseases, Center of Advanced Pharmaceuticals and Biomaterials, China Pharmaceutical University, Nanjing 210009, PR China.

^6^State Key Laboratory of Pathogenesis, Prevention and Treatment of High Incidence Diseases in Central Asia, School of Medical Engineering and Technology & Technology Innovation and Translational Service Center, Xinjiang Medical University, Urumqi 830054, P. R. China.

^*^Address correspondence to: Nuernisha Alifu; [nens_xjmu@126.com](mailto:nens_xjmu@126.com), Cheng Zhou; [czhou@scut.edu.cn](mailto:czhou@scut.edu.cn) and Zonghai Sheng; [zh.sheng@siat.ac.cn](mailto:zh.sheng@siat.ac.cn)

†These authors contributed equally to this work.

**
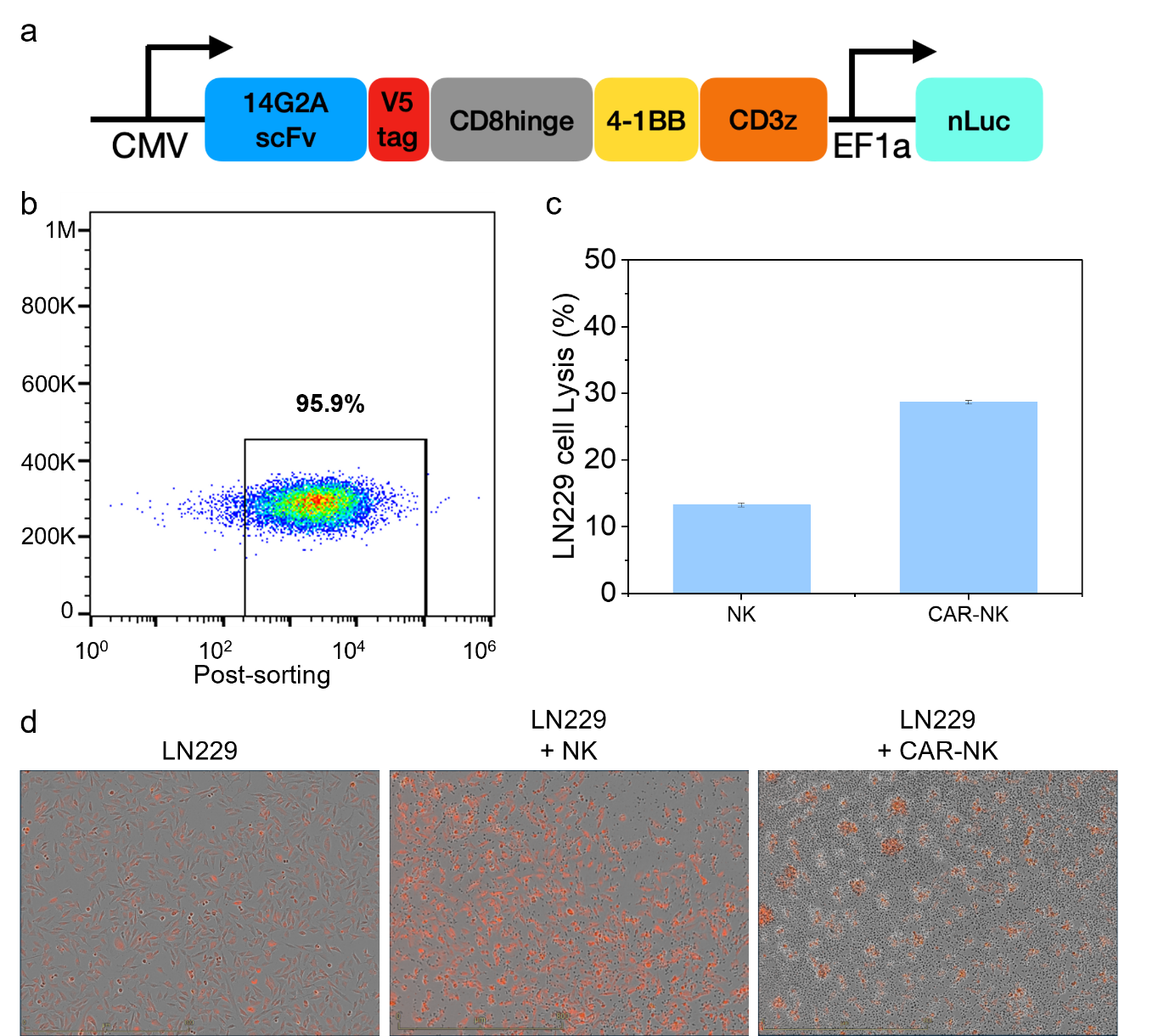
**

**Figure S1: Generation and characterization of CAR-NK cells. a.** Structure of GD2 CAR nLuc Lentiviral vector. **b.** Flow cytometry analysis and sorting of CAR-expressing NK cells. **c.** Cell lysis ratio of LN229 cells co-cultured with NK and CAR-NK cells at the time point of 18 hours. **d.** Brightfield microscopy imaging of LN229 cells co-cultured with Celltrace Far Red dye-labeled NK and CAR-NK cells.


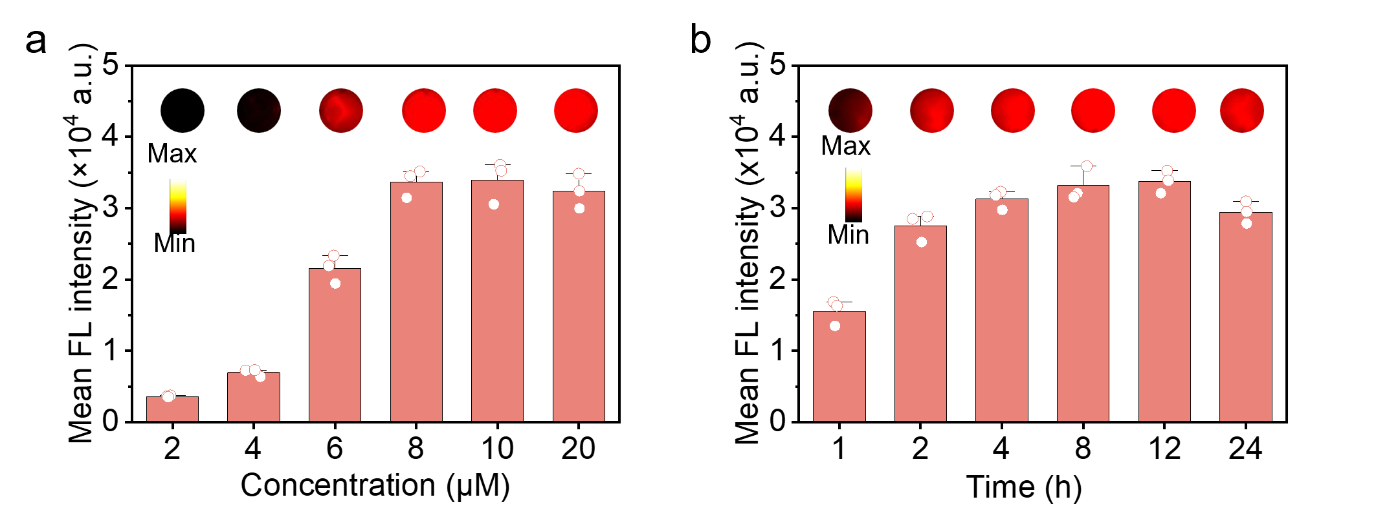


**Figure S2. Optimization of CAR-NK labeling with COE-BBT.** NIR-II fluorescence image (λ_exc_ = 808 nm) and the mean fluorescence (FL) intensity quantification of a test well filled with CAR-NK cells, labeled with COE-BBT probe in varied incubation concentration (**a.**) or time (**b,**). Images captured utilizing a 1200 nm long-pass filter, 1500 ms exposure time. Data represent mean ± SD, n = 3.

**
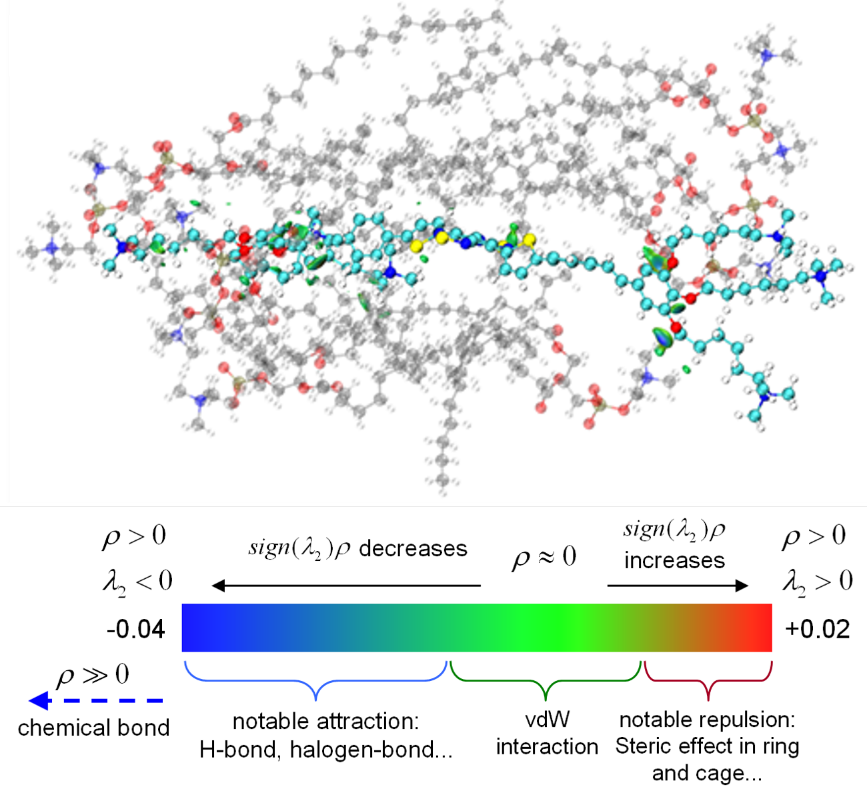
**

**Figure S3. Independent Gradient Model (IGM) analysis of COE-BBT cellular membrane insertion**

**
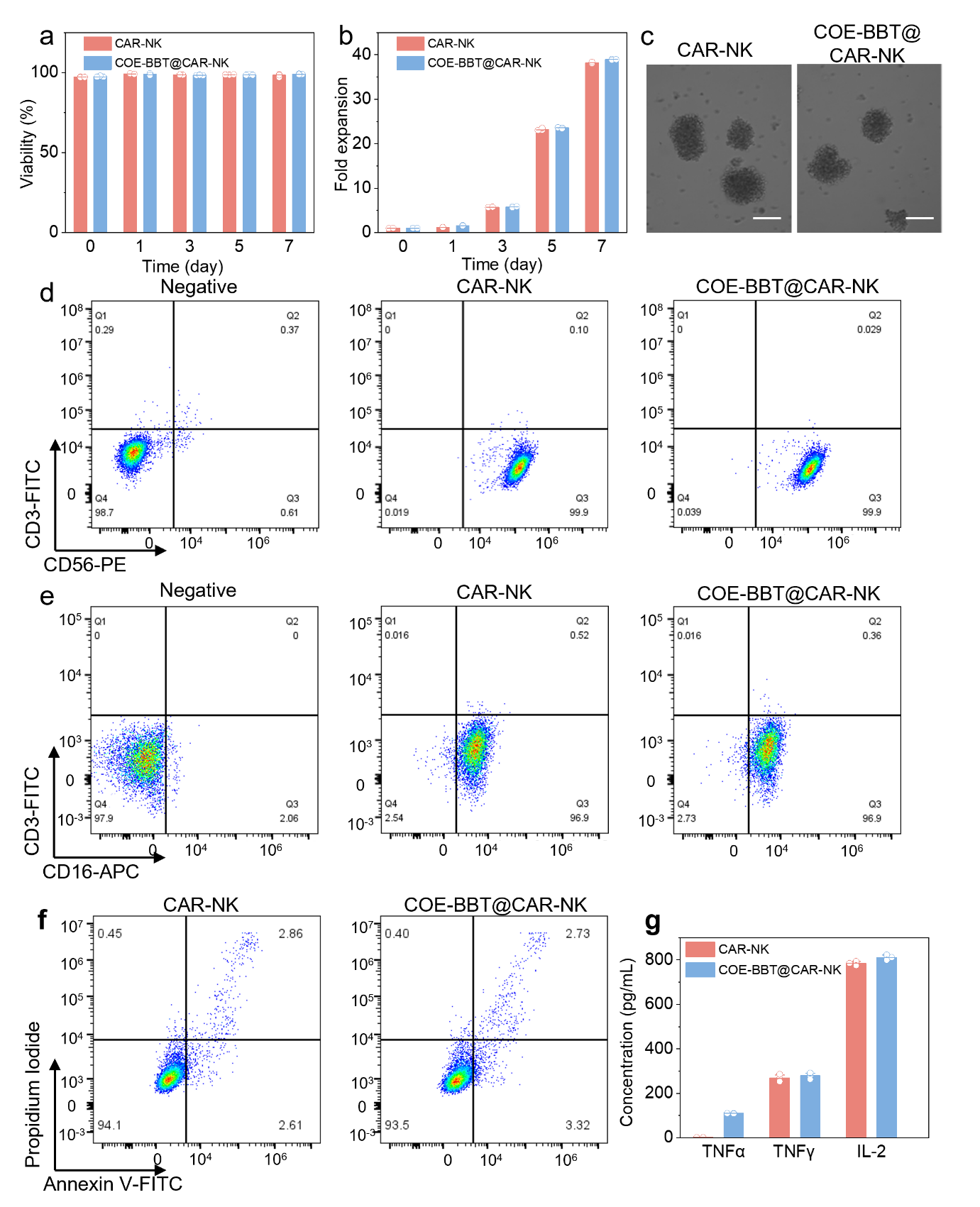
**

**Figure S4. Function characterization of COE-BBT labeled CAR-NK cells. a.** Cell viability and **b.** the proliferation ability of unlabeled and COE-BBT-labeled CAR-NK cells were compared upon trypan blue staining. **c.** Bright-field imaging of CAR-NK cells with and without COE-BBT labeling. Scale bar, 1 mm. **d. e.** Flow cytometry analysis of CD3, CD56 (d.), and CD16 (e.) to determine CAR-NK cell phenotype upon COE-BBT labeling. **f.** Flow cytometry analysis to assess CAR-NK cell apoptosis upon COE-BBT labeling. **g.** ELISA measurement of cytokines TNF-α, IFN-γ, and IL-2 secreted by CAR-NK cells upon COE-BBT labeling. Data represent mean ± SD, n = 3.

**
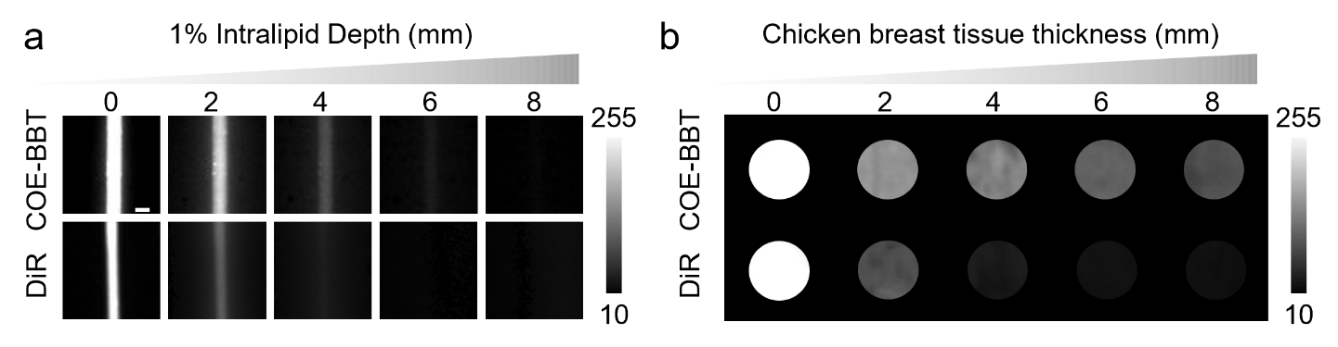
**

**Figure S5. Penetration depth of COE-BBT@CAR-NK cells in comparison to DiR labeling. a.** NIR-II fluorescence images of a glass capillary filled with CAR-NK cells labeled with COE-BBT or DiR in 1% intralipid vatied in depth (0, 2, 4, 6, and 8 mm). **b.** NIR-II fluorescence images of CAR-NK cells labeled with COE-BBT or DiR in a 96-well plate and covered with chicken breast tissue varied in thickness (0, 2, 4, 6, and 8 mm). λ_exc_ = 808 nm. Images captured utilizing a 1000 nm long-pass filter, 1200 ms exposure time.

**
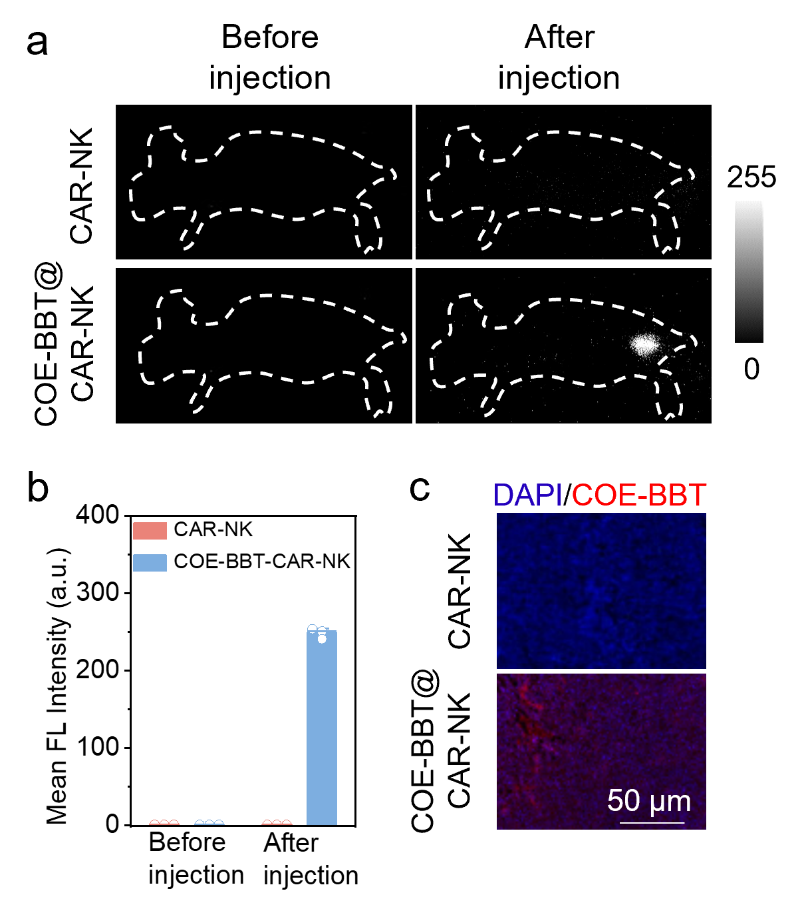
**

**Figure S6. Capability of CAR-NK in vivo imaging. a.** NIR-II fluorescence images (λ_exc_ = 808 nm) and **b.** mean FL intensity quantification of nude mice subcutaneously injected with COE-BBT@CAR-NK with varied cell numbers. Images captured utilizing a 1000 nm long-pass filter, 500 ms exposure time. Data represent mean ± SD, n = 3. **c.** Confocal images of tumor sections (nuclei: blue) post-injected with COE-BBT@CAR-NK cells (red). Scale bar = 50 μm.

**
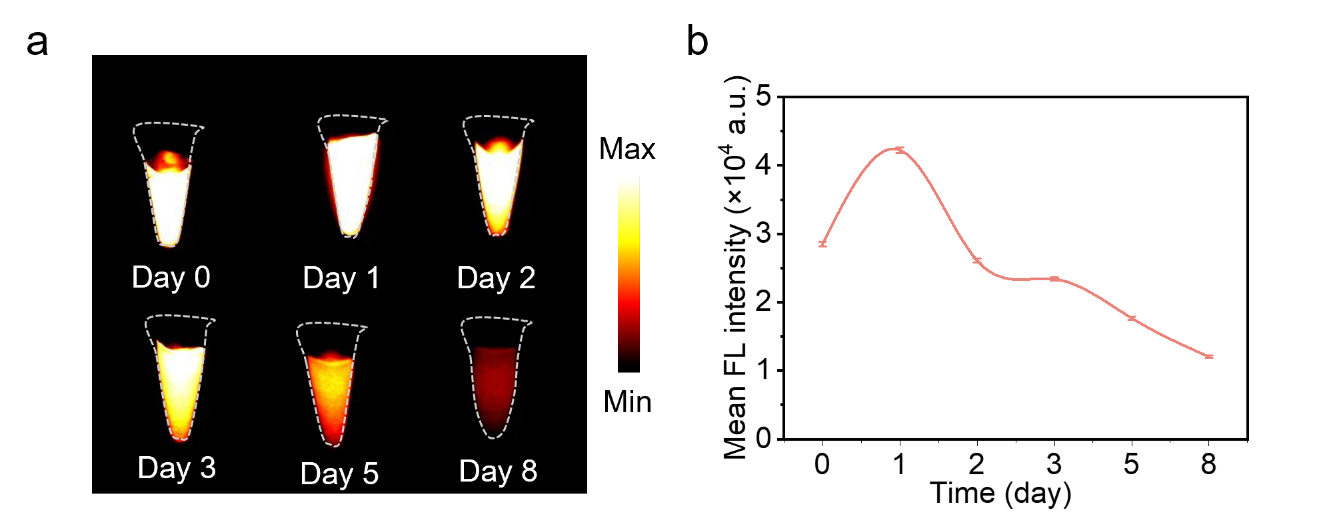
**

**Figure S7. Optimization of CAR-NK labeling with COE-BBT. a.** NIR-II fluorescence images (λ_exc_ = 808 nm) and **b.** the mean fluorescence (FL) intensity quantification of a test tube filled with COE-BBT@CAR-NK cells on day 0, 1, 2, 3, 5, and 8 post COE-BBT staining. Images captured utilizing a 1200 nm long-pass filter, 1500 ms exposure time. Data represent mean ± SD, n = 3.

**
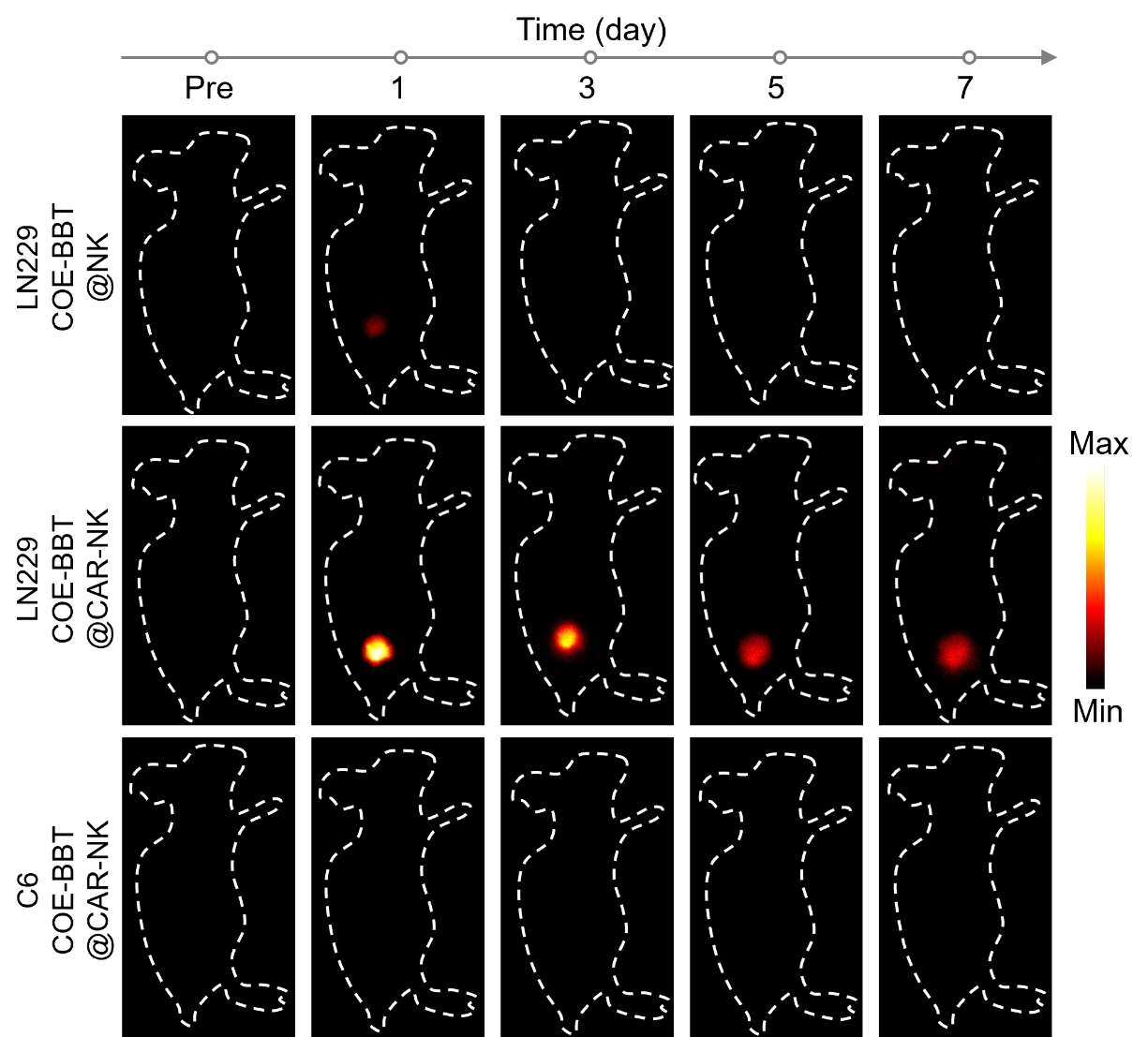
**

**Figure S8. Subcutaneous tumor homing of COE-BBT@CAR-NK cells.** NIR-II fluorescence imaging (λ_exc_ = 808 nm) of LN229 and C6 subcutaneous glioma-bearing mice at various time points post COE-BBT@CAR-NK and COE-BBT@NK cell intravenous injection. Images captured utilizing a 1000 nm long-pass filter, 500 ms exposure time.


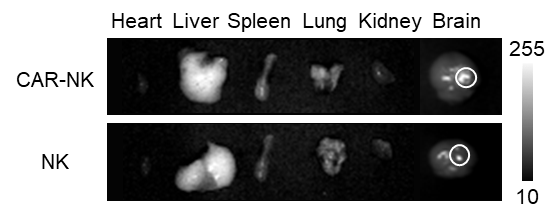


**Figure S9. Major organ imaging post intravenous injection of COE-BBT labeled cells.** NIR-II fluorescence imaging (λ_exc_ = 808 nm) of *ex vivo* major organs (heart, liver, spleen, lung, kidney, and brain) of glioma-bearing mice post COE-BBT@CAR-NK and COE-BBT@NK cell intravenous injection. Images captured utilizing a 1000 nm long-pass filter, 500 ms exposure time.

**
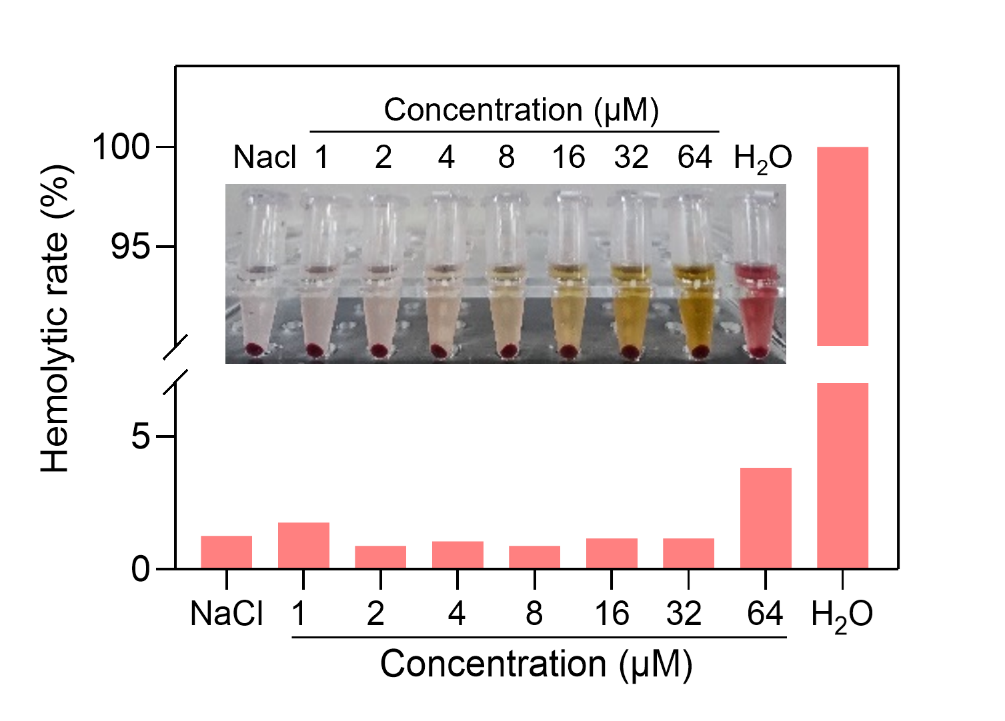
**

**Figure S10. Hemolytic analysis of COE-BBT.** Hemolysis of mouse erythrocytes in the COE-BBT probe at different concentrations was evaluated and normalised to the H_2_O group, which served as the positive control.

**
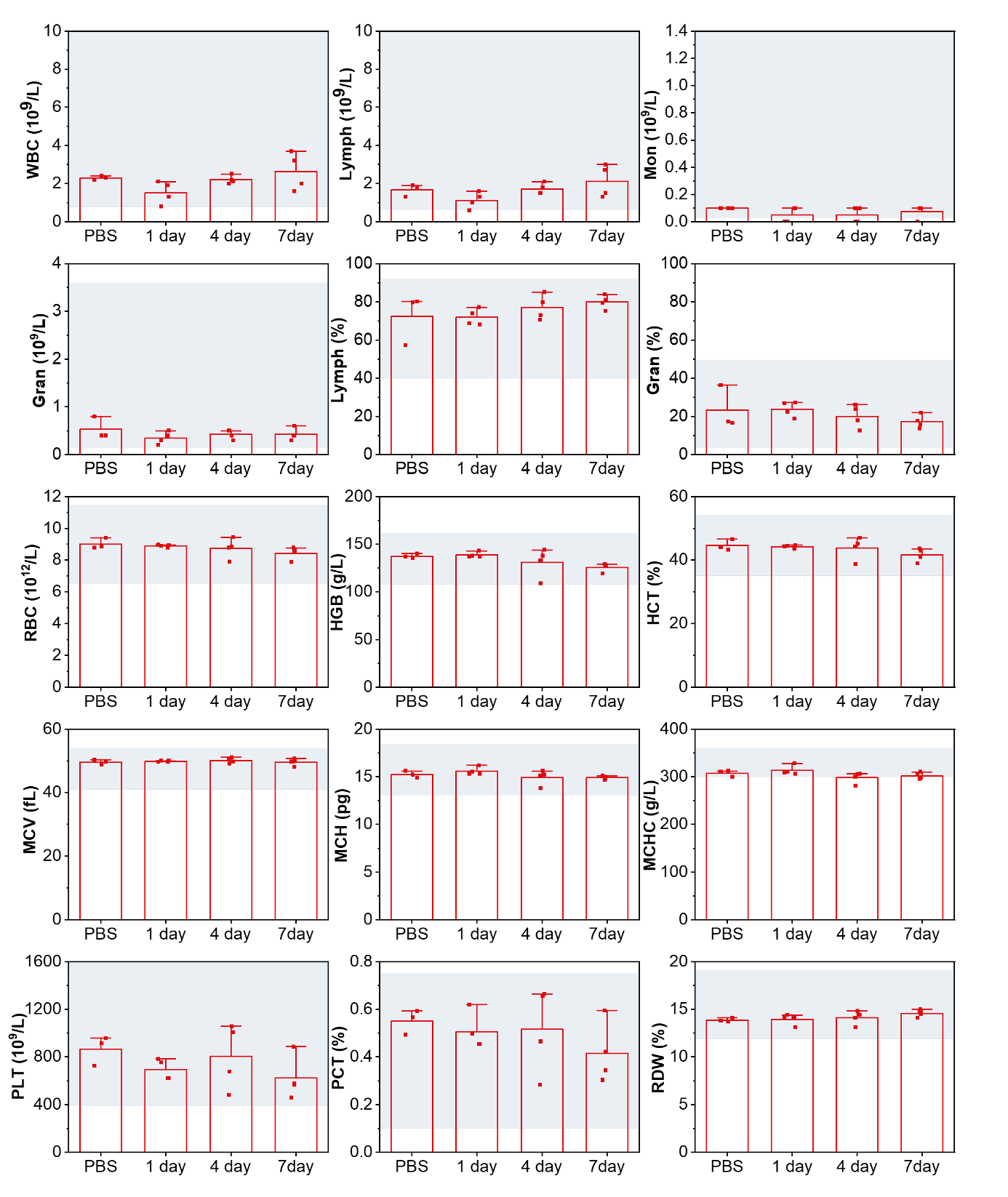
**

**Figure S11. Blood routine analysis of COE-BBT@CAR-NK.** Effects of COE-BBT@CAR-NK cells on immune response, oxygen transport capacity, and coagulation function. Abbreviations and reference range: White Blood Cell: WBC (0.8–10.6 ×109/L ); Lymphocyte: Lymph (0.6–8.9 ×109/L); Monocyte: Mon (0.04–1.4 ×109/L) Granulocyte: Gran (0.23–3.6 ×109/L); Lymphocyte Percentage: Lymph (40–92%); Granulocyte Percentage: Gran (6.5–50%); Red Blood Cell: RBC (6.5–11.5 ×1012/L); Hemoglobin: HGB(110–165 g/L); Hematocrit Percentage: HCT (35–55%) Mean Corpuscular Volume: MCV (41–55 fL); Mean Corpuscular Hemoglobin: MCH (13–18 pg) Mean Corpuscular Hemoglobin Concentration: MCHC (300–360 g/L) Platelet: PLT (400–1600 ×109/L); Plateletcrit Percentage: PCT (0.100–0.780%); Red Blood Cell Distribution Width Percentage: RDW(12–19%). Data represent mean ± SD, n = 4.

**
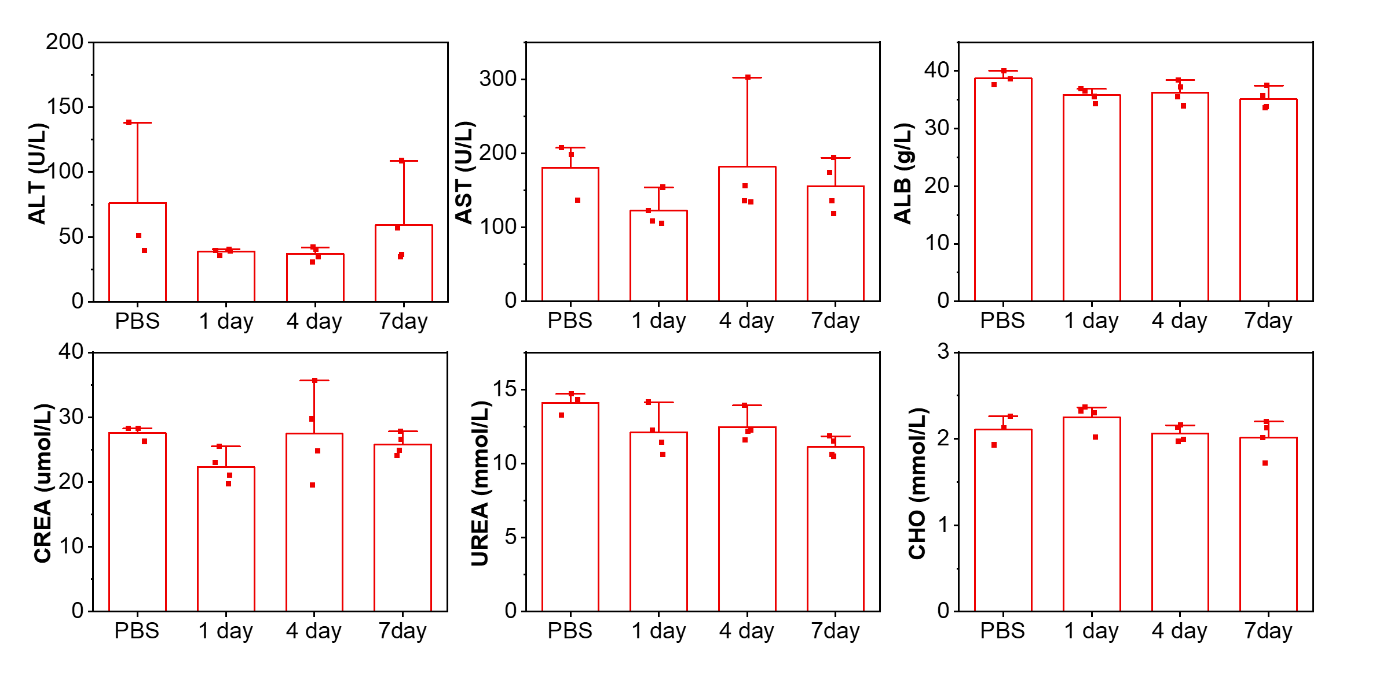
**

**Figure S12. Biochemical analysis of COE-BBT@CAR-NK.** Abbreviations: Alanine aminotransferase: ALT; aspartate aminotransferase: AST; albumin: ALB; creatinine: CREA; urea: UREA; cholesterol: CHO. Data represent mean ± SD, n = 4.


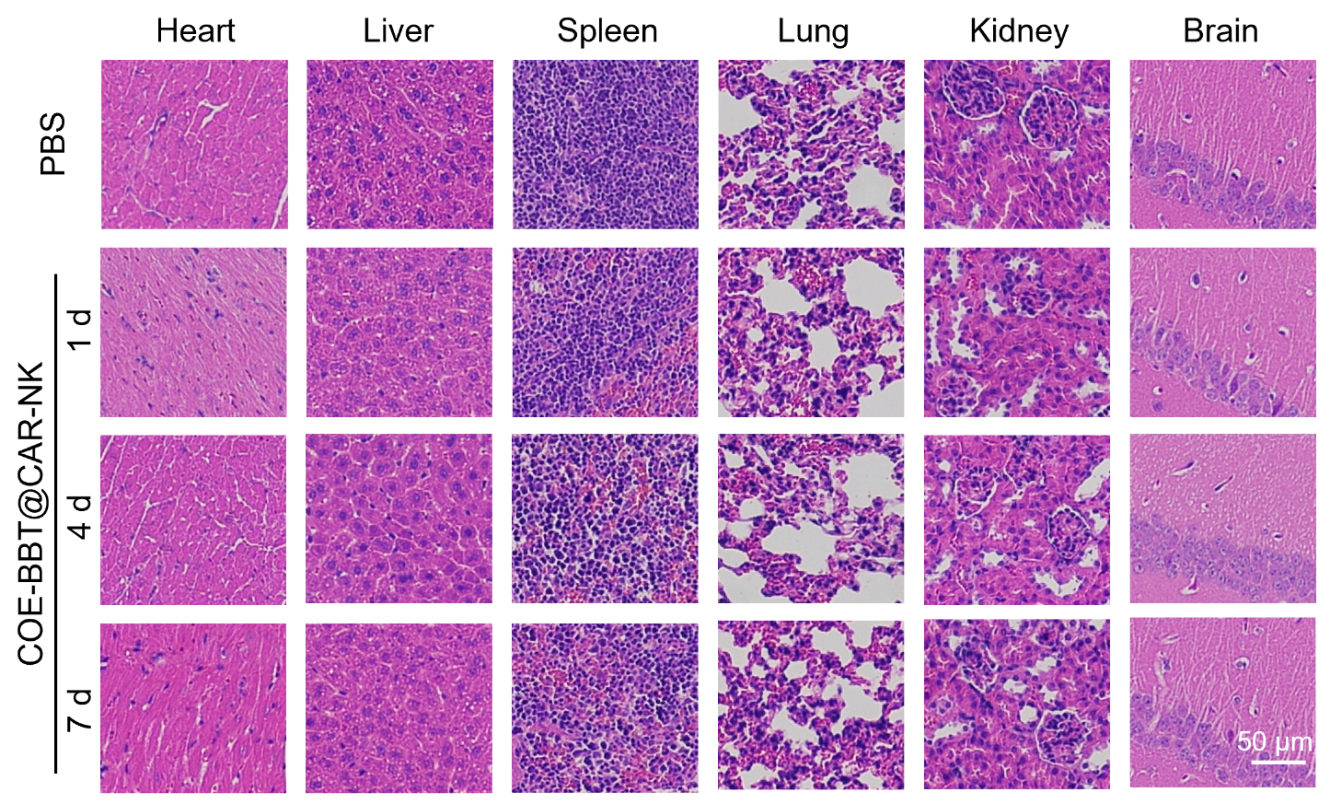


**Figure S13. Biochemical analysis of COE-BBT@CAR-NK.** Abbreviations: Alanine aminotransferase: ALT; aspartate aminotransferase: AST; albumin: ALB; creatinine: CREA; urea: UREA; cholesterol: CHO. Data represent mean ± SD, n = 4.
